# MultiMS2: A curated multi-modal, multi-energy spectral library for metabolomics

**DOI:** 10.1093/gigascience/giag069

**Published:** 2026-06-10

**Authors:** Adriano Rutz, Mario S P Correia, Nicola Zamboni

**Affiliations:** Institute for Molecular Systems Biology, ETH Zürich, Otto-Stern-Weg 3, 8093 Zürich, Switzerland; Institute for Molecular Systems Biology, ETH Zürich, Otto-Stern-Weg 3, 8093 Zürich, Switzerland; Institute for Molecular Systems Biology, ETH Zürich, Otto-Stern-Weg 3, 8093 Zürich, Switzerland

**Keywords:** spectral library, collision-induced dissociation, electron-activated dissociation, metabolomics

## Abstract

**Background:**

Spectral libraries are essential for mass spectrometry-based metabolomics, enabling accurate metabolite annotation. Collision-induced dissociation (CID) dominates existing public libraries, but is rarely sufficient for structural elucidation. Electron-activated dissociation (EAD) provides complementary, radical-driven fragmentation, but remains sparsely represented. The lack of datasets spanning multiple dissociation mechanisms, energies, and ionization modes limits both analytical workflows and the development of robust machine learning models.

**Findings:**

We present MultiMS^2^, a curated metabolomics spectral library comprising 43,728 MS/MS spectra from 2,899 unique compounds. Spectra were acquired using both CID and EAD at three energies each, in positive and negative ionization modes. The dataset substantially expands publicly available EAD coverage while preserving matched acquisition conditions across energies and dissociation types.

**Conclusions:**

By systematically combining CID and EAD across multiple energies and polarities, MultiMS^2^ provides a unique resource for metabolite annotation, benchmarking, and machine learning. The library supports energy-aware and dissociation-aware analysis, enabling methodological innovation and improved generalization in computational metabolomics.

## Key points

- Comprehensive spectral library for metabolomics spanning three energies and both polarities.
- Includes both collision-induced and electron-activated dissociation, greatly expanding EAD coverage.
- Enables improved metabolite annotation, machine learning, and method development.

## Context

Metabolomics relies heavily on tandem mass spectrometry (MS^2^) to characterize and annotate small molecules in biological systems. Confident metabolite annotation typically depends on comparison to reference spectral libraries. In recent years, machine learning has emerged as a central approach for automated annotation, spectrum prediction, and structure elucidation, but its success depends critically on access to large, diverse, and well-annotated training datasets.

Most existing public metabolomics libraries are dominated by collision-induced dissociation (CID) spectra. While CID is robust and widely available, it often favors low-energy fragmentation pathways and may miss structurally informative cleavages. In contrast, electron-activated dissociation (EAD) generates complementary radical-driven fragment ions that can enhance structural elucidation. Despite this potential, EAD spectra remain scarce in public repositories, restricting both manual interpretation and the ability of machine learning models to generalize across fragmentation mechanisms.

The accessibility and standardization of MS^2^ data has substantially advanced thanks to long-standing community repositories like GNPS [1] or MassBank [2] and its North American version (RRID:SCR_015536), harmonization efforts in large-scale MS/MS library curation [3,4], and large individual initiatives [5–7]. However, these resources typically lack systematic coverage across dissociation mechanisms and multiple energies for the same set of compounds, leaving an important gap for workflows and computational models requiring broad fragmentation diversity. Further comprehensive MS fragmentation libraries such as METLIN [8] or NIST (RRID:SCR_014668) exist, but are not openly accessible.

To address this gap, we present MultiMS^2^, a curated spectral library that systematically combines CID and EAD across three energies in both positive and negative ionization modes. Through rigorous curation and quality control, this resource aims to improve metabolite annotation and to provide a benchmark dataset for developing and evaluating machine learning methods that are robust to fragmentation physics and acquisition conditions.

## Methods

### Experimental

We analyzed three libraries of pure chemical standards. First, the Human Endogenous Metabolite Compound Library (ca. 1,000 standards; Selleck Chemicals, Art. No. L4500), which was pooled in sets of 10 compounds and diluted with 10% (v/v) ethanol to a final concentration of 10 µM for injection. Second, the Mass Spectrometry Metabolite Library (MSMLS; Merck, Art. No MSMLS-1EA, Lot 2016), which was dissolved and diluted according to the manufacturer instructions (water for plates 1–5, methanol for plates 6–7). Compounds were pooled in sets of 10 and diluted to a final concentration of 5–20 µM. Third, a library of ca. 3,000 natural product-like compounds was obtained from NEXUS, the chemical screening facility of our institution, pre-pooled in sets of 10. Compounds were diluted with 10% (v/v) ethanol to a final concentration of 10 µM. Overall, compounds were used as supplied by the manufacturer (pre-dissolved) or prepared following manufacturer protocols. Compound pooling was designed to maximize throughput; pools were assembled to minimize precursor mass overlap, and any remaining conflicts were resolved during data processing, consistent with standard practice in large-scale spectral library acquisition workflows.

Spectra were acquired using a SCIEX ZenoTof 7,600 System coupled to an Agilent Infinity II LC stack. Direct injection (5 µl) was performed using a mobile phase made of 50:50 mixture of water:methanol (both containing 0.1% formic acid) with a flow rate of 0.2 ml/min. TOFMS data were acquired from 50 to 1,500 *m/z* with an accumulation time of 50 ms, declustering potential of 50 V, collision energy of 10 V, curtain gas at 45 (arbitrary units), CAD gas at 7 (arbitrary units), ion source gas 1 and 2 at 70 psi, source temperature at 700°C, and a spray voltage of 5,500 V for positive mode and −4,500 V for negative mode. Information-dependent acquisition (IDA) selected up to two ions per cycle for MS/MS, with dynamic background subtraction enabled. Zeno pulsing was applied with a threshold of 20,000 cps. Precursor ions were targeted with a mass tolerance of 50 mDa and an exclusion window of 2 s. Three collision energies were set for CID (20, 40, and 60 V) and EAD, respectively (12, 16, and 24 electron kinetic energy, with a current of 3,500 V and 30 ms activation time). The total method duration was 0.6 min (actual acquisition time 1.06 min), with 188 estimated cycles per run.

### Data processing

Raw *.wiff* data were converted to profile *.mzML* using ProteoWizard (v3.0.25182) (RRID:SCR_012056). Centroiding was performed using CentroidR (v0.0.0.9001) [9]. Spectral library was built using mzmine (v.4.7.27) (RRID:SCR_012040) and custom Python programs (RRID:SCR_008394) (archived at Zenodo [RRID:SCR_004129 ]) [10]. Annotations include SMILES [11], InChI and InChIKeys [12], and SELFIES representations [13], along with complete instrument and acquisition metadata. Spectra are distributed in mzML and MGF formats with accompanying metadata tables.

## Data validation and quality control

The spectra were inspected using a combination of automated and manual quality control procedures to ensure correct precursor assignment, spectral purity, and annotation accuracy. From the initial 148,888 candidate spectra collected, thresholds for precursor purity and spectral quality were applied uniformly across modalities, and all retained spectra passed these criteria. A minimal precursor height of 1,000 counts was required together with a minimal precursor purity of 0.9. To be retained, spectra had to be present in at least two modalities. The minimal number of fragments was set to 3, with at least 5% explained signals and 40% explained intensity. If multiple spectra per modality were left, only the ones with at least 40% of the maximal explained signals and 80% of the maximal explained intensity were kept. This allowed us to keep multiple replicates per modality while ensuring quality. Key dataset statistics are summarized in Table 1. Representative results and validation workflows are documented in [14] and archived at [10].

**Table 1:** Key statistics of the MultiMS^2^ spectral library.

Item	Quantity
Unique compounds	2,899 *
Unique compound-adduct modalities	4,210
Unique compound-adduct-fragmentation modalities	17,170
Unique spectra	43,728

* As defined by the connectivity information encoded in the first 14 characters of the corresponding InChIKey (see [12]).

**Figure 1:**
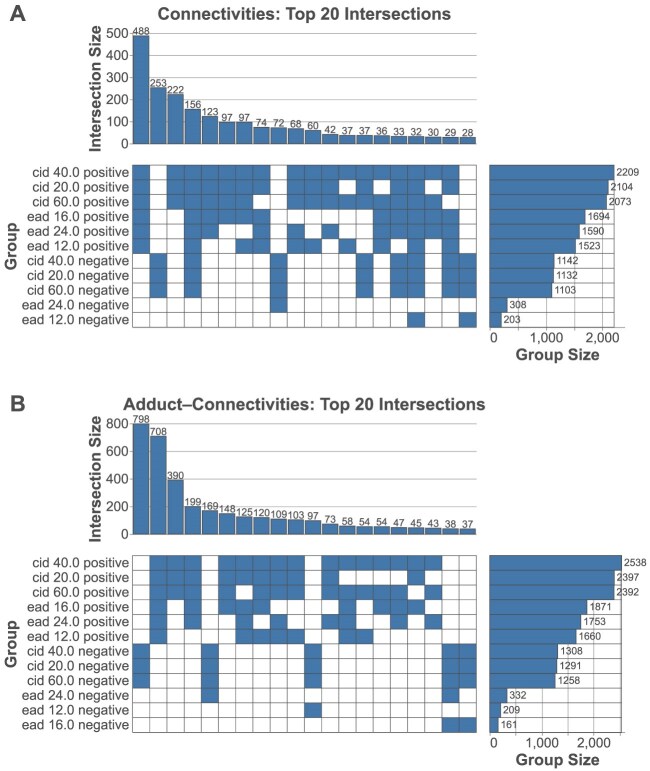
Overlaps between modalities. Total group sizes are shown on the right, and intersection sizes at the top. Only the top 20 intersections are displayed. Panel A: Overlap of compounds fragmented across modalities (e.g., 676 compounds (488 + 156 + 32) in all positive modalities). Panel B: Overlap of compound–adduct pairs, considering both molecular ion and adduct type. Trends mirror Panel A, except no adduct types are shared between negative and positive ionizations.

Figure 1 shows modality overlaps using upset plots, complementing the absolute counts in Table 1 by revealing the actual extent of feature sharing; specifically, shared compound identities (Panel A) and compound–adduct pairs (Panel B). Panel A reveals strong ionization-mode specificity: the two largest intersections correspond to compounds detected exclusively in positive or negative ionization, underscoring the chemical selectivity of each mode. The fourth-largest intersection (156 compounds) includes features detected across all positive modalities except negative electron-activated dissociation (EAD), consistent with the known limitations of EAD for anions, where low electron affinity and poor fragmentation efficiency reduce detection coverage. An additional reason for the low yield of MS spectra for negative EAD is the use of formic acid as solvent modifier. Albeit the good recovery of MS spectra for negative CID suggests that its impact on ESI efficiency of anions is not dramatic, the effect might be more important on the generally less abundant EAD fragments. Future library updates will explore the use of alkaline modifiers such as ammonium hydroxide to improve negative mode coverage. Ongoing methodological improvements aim to address this gap [15]. In total, 676 compounds (488 + 156 + 32) were consistently detected across all positive-mode modalities.

Spectral quality was assessed using MSBuddy [16], one of the few tools explicitly designed to account for radical-driven fragmentation in subformula assignment. This is a critical feature for evaluating EAD spectra, where unpaired electrons dominate dissociation pathways. Unlike conventional tools optimized for even-electron CID fragmentation, MSBuddy does not penalize spectra with odd-electron fragments, making it better suited for cross-modal comparison.

**Figure 2:**
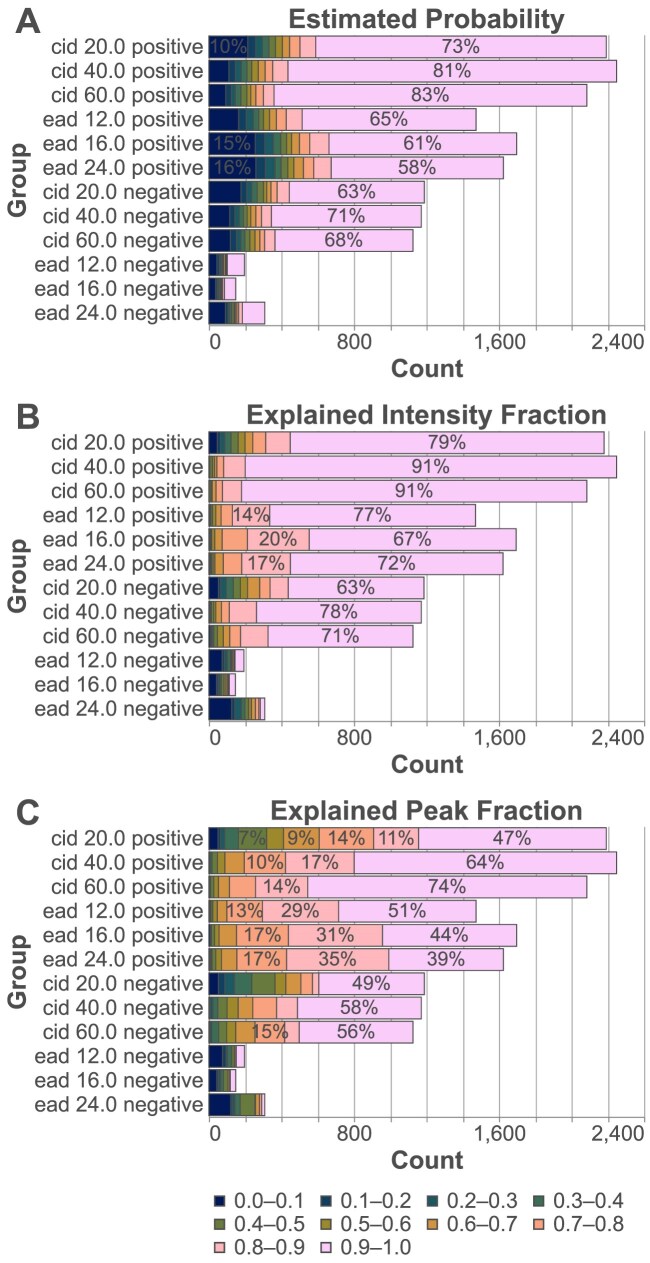
Spectral quality metrics from BUDDY. Some of the metrics calculated by BUDDY were used as proxies to assess spectral quality. Panel A: Estimated probability of the assigned molecular formula. Overall, calculated probabilities were high (around 80% above 0.9 for CID positive). Probabilities increased with higher CID energy but decreased for EAD. Probabilities were lower in negative mode. Panel B: Fraction of total MS intensity explained by subformulae. Similar to previous panel, the proportion of spectra considered high-quality by this metric was generally high. Panel C: Fraction of total fragment count explained by subformulae. This complements Panel B, since a single very intense ion could otherwise bias the interpretation.

As shown in Fig. 2, CID spectra yielded higher average molecular formula assignment probabilities compared to EAD spectra. Similarly, the fraction of fragment intensity explained by assigned subformulae was higher for CID. While these differences reflect the inherent complexity of radical-mediated fragmentation in EAD, the different information within the spectrum might help for finer structural elucidation and not particularly for formula determination. The slightly lower scores for EAD may also reflect the presence of multicharged ions, which are more prevalent in EAD spectra. Finally, on all A, B, and C panels, increasing fragmentation energy was beneficial for CID while detrimental for EAD.

**Figure 3:**
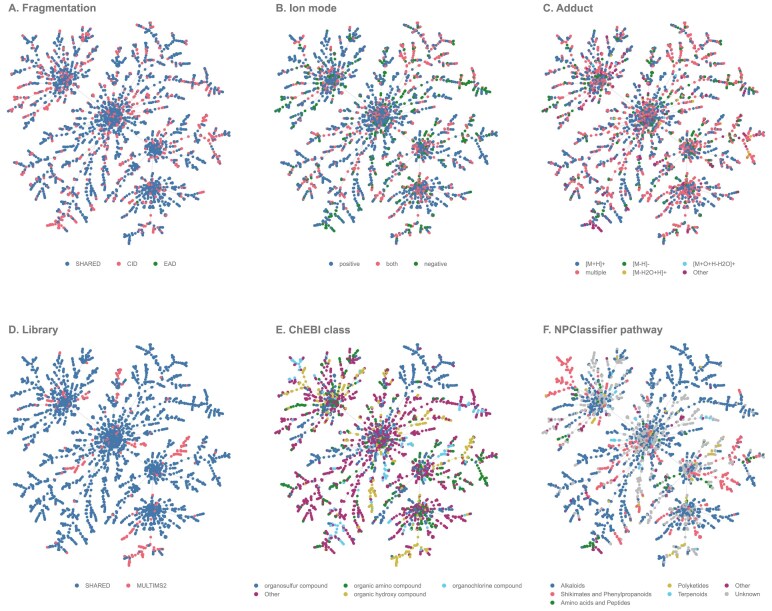
Chemical space coverage of the MultiMS^2^ library. Each node represents a unique compound; layout reflects structural similarity based on MAP4 fingerprints [17] embedded via MinHash LSH and visualized with TMAP [18]. The same layout is colored by six metadata dimensions. The upper row represents metadata from the dataset itself while the lower one represents external metadata. An interactive version is available in the project repository [14]. Panel A: Fragmentation mode. CID-only, EAD-only, and shared modalities, with the majority of the compounds acquired in both modalities. Panel B: Ionization mode. Positive-only, negative-only, and compounds detected in both modes. Panel C: Adduct type. The dominant adduct *[M + H]^+^* accounts for most positive-mode entries; *[M − H]^−^* dominates negative mode; but a great variety of less common adducts is also visible. Panel D: Spectral libraries. Comparison with openly accessible external spectral libraries. Some clustered areas were previously not covered, confirming that MultiMS^2^ provides substantial novel coverage. Panel E: ChEBI chemical class [19]. Showing the chemical diversity of the library. Panel F: NPClassifier biosynthetic pathway [20]. Alkaloids, shikimates and phenylpropanoids, amino acids and peptides, polyketides, and terpenoids are well represented.

To visualize the chemical diversity of the MultiMS^2^ library, we embedded all compounds using MinHash fingerprints (MAP4) [17] and constructed a Tree MAP (TMAP) [18] (Fig. 3). Each node represents a unique compound; proximity reflects structural similarity. The resulting map was annotated with six orthogonal metadata layers to assess how acquisition properties and chemical classifications distribute across chemical space. Panel A shows the proportion of spectra obtained in CID-only, EAD-only or in both modalities. Similarly, ionization mode (Panel B) and adduct type (Panel C) show a good coverage of different modalities per compound. Panel D shows the overlap of MultiMS^2^ entries with all openly accessible spectral libraries, with some clusters only present in MultiMS^2^. Panels E and F reveal that ChEBI chemical classes (computed using [19]) and NPClassifier (NPC) biosynthetic pathway assignments [20] are also diverse. Taken together, the TMAP visualization demonstrates that MultiMS^2^ provides broad and structurally diverse coverage, spanning multiple compound classes and biosynthetic families, while maintaining balanced representation across the acquisition conditions central to this library.

## Re-use potential

MultiMS^2^ significantly enhances metabolite annotation in both untargeted and targeted metabolomics by offering systematic, multidimensional coverage of dissociation mechanisms, collision energies, and ionization polarities. This structured design makes it uniquely suited for training and evaluating machine learning models, particularly for tasks such as:

- **Fragmentation prediction**: Modeling how molecules break under varying conditions.
- **Energy-aware modeling**: Incorporating collision energy as a continuous variable to improve spectral simulation.
- **Cross-dissociation transfer learning**: Leveraging knowledge from one fragmentation technique to improve performance on another.

The dataset also serves as a benchmark for model robustness, enabling direct comparison of algorithm performance across different fragmentation techniques.

Beyond machine learning, MultiMS^2^ can be integrated into existing spectral matching platforms, enhancing annotation confidence through multi-modal spectral libraries. It supports workflows in environmental screening, clinical metabolomics, and systems biology.

## Availability of source code and requirements

- Project name: MultiMS^2^
- Project repository: https://github.com/zamboni-lab/MultiMS2
- Operating system(s): Platform independent (Docker container provided)
- Programming language: Python Programming Language (RRID:SCR_008394) R Project for Statistical Computing (RRID:SCR_001905) Bash (RRID:SCR_021268)
- Other requirements: Docker Desktop (RRID:SCR_016445) ProteoWizard (RRID:SCR_012056) mzmine (RRID:SCR_012040) uv (https://docs.astral.sh/uv/)
- License: MIT License
- Any restrictions to use by non-academics: None

## Abbreviations

CID: collision-induced dissociation; EAD: electron-activated dissociation; InChI(Key): international chemical identifier (key); MassIVE: mass spectrometry interactive virtual environment; SELFIES: self-referencing embedded strings; SMILES: simplified molecular input line entry system.

## Supplementary Material

giag069_Authors_Response_To_Reviewer_Comments_original_submission

giag069_Authors_Response_To_Reviewer_Comments_revision_1

giag069_GIGA-D-25-00518_original_submission

giag069_GIGA-D-25-00518_revision_1

giag069_GIGA-D-25-00518_revision_2

giag069_Reviewer_1_Report_original_submission Reviewer 1 -- 1/13/2026

giag069_Reviewer_2_Report_original_submission Reviewer 2 -- 1/25/2026

giag069_Reviewer_2_Report_revision_1 Reviewer 2 -- 4/27/2026

giag069_Reviewer_2_Report_revision_2 Reviewer 2 -- 5/15/2026

## Data Availability

The data sets supporting the results of this article are available in both Zenodo [21] and MassIVE repositories [22] under the permissive CC0 1.0 Universal License. Because of size limitations, the library was split into five partitions on GNPS [23–27].
