# Supplementary material for: MultiMS2: A curated multi-modal, multi-energy spectral library for metabolomics
Source: Gigascience. 2026 Jun 10;15:giag069. doi: 10.1093/gigascience/giag069 (PMC13312951; doi:10.1093/gigascience/giag069)
Supplement: giag069_GIGA-D-25-00518_original_submission [file giag069_giga-d-25-00518_original_submission.pdf]

GigaScience

MultiMS2: A Curated Multi-Modal, Multi-Energy Spectral Library for Metabolomics

--Manuscript Draft--

**Manuscript Number:**	GIGA-D-25-00518
**Full Title:**	MultiMS2: A Curated Multi-Modal, Multi-Energy Spectral Library for Metabolomics
**Article Type:**	Data Note
**Funding Information:**	Schweizerischer Nationalfonds zur Förderung der Wissenschaftlichen Forschung (10002786) — Prof. Nicola Zamboni
**Abstract:**	Background:<br>Spectral libraries are essential for mass spectrometry-based metabolomics, enabling accurate metabolite annotation. Collision-induced dissociation (CID) dominates existing public libraries, but is rarely sufficient for structural elucidation. Electron-activated dissociation (EAD) provides complementary, radical-driven fragmentation, but remains sparsely represented. The lack of datasets spanning multiple dissociation mechanisms, energies, and ionization modes limits both analytical workflows and the development of robust machine learning models.<br>Findings:<br>We present MultiMS2, a curated metabolomics spectral library comprising 43,728 MS/MS spectra from 2,899 unique compounds. Spectra were acquired using both CID and EAD at three energies each, in positive and negative ionization modes. The dataset substantially expands publicly available EAD coverage while preserving matched acquisition conditions across energies and dissociation types.<br>Conclusions:<br>By systematically combining CID and EAD across multiple energies and polarities, MultiMS2 provides a unique resource for metabolite annotation, benchmarking, and machine learning. The library supports energy-aware and dissociation-aware analyses, enabling methodological innovation and improved generalization in computational metabolomics.
**Corresponding Author:**	Nicola Zamboni<br>ETH Zürich D-BIOL: Eidgenossische Technische Hochschule Zurich Departement Biologie<br>Zurich, SWITZERLAND
**Corresponding Author Secondary Information:**	
**Corresponding Author's Institution:**	ETH Zürich D-BIOL: Eidgenossische Technische Hochschule Zurich Departement Biologie
**Corresponding Author's Secondary Institution:**	
**First Author:**	Adriano Rutz
**First Author Secondary Information:**	
**Order of Authors:**	Adriano Rutz<br>Mario Sergio Povoa Correia<br>Nicola Zamboni
**Order of Authors Secondary Information:**	
**Additional Information:**	
**Question**	**Response**
Are you submitting this manuscript to a special series or article collection?	No

Powered by Editorial Manager® and ProduXion Manager® from Aries Systems Corporation

**Experimental design and statistics**<br><br>Full details of the experimental design and statistical methods used should be given in the Methods section, as detailed in our Minimum Standards Reporting Checklist. Information essential to interpreting the data presented should be made available in the figure legends.<br><br>Have you included all the information requested in your manuscript?	Yes
**Resources**<br><br>A description of all resources used, including antibodies, cell lines, animals and software tools, with enough information to allow them to be uniquely identified, should be included in the Methods section. Authors are strongly encouraged to cite Research Resource Identifiers (RRIDs) for antibodies, model organisms and tools, where possible.<br><br>Have you included the information requested as detailed in our Minimum Standards Reporting Checklist?	Yes
**Availability of data and materials**<br><br>All datasets and code on which the conclusions of the paper rely must be either included in your submission or deposited in publicly available repositories (where available and ethically appropriate), referencing such data using a unique identifier in the references and in the “Availability of Data and Materials” section of your manuscript.<br><br>Have you have met the above requirement as detailed in our Minimum Standards Reporting Checklist?	Yes

GigaScience has policies and guidelines in place for the use of generative AI-writing tools such as ChatGPT. If you have used such writing tools to assist with writing the manuscript this must be declared and cited in the text. Authors should not list AI-writing tools and other AI-assisted technologies as an author or co-author and should acknowledge that they are fully responsible for text generated or refined by AI-writing tools.<p><br><br>A summary of use (particularly in the introduction or among methods) needs to be included at the end of the paper, and the outputs should also be included as a supplementary file hosted in GigaDB or other open repositories. Please read our guidelines for more information. <p><br><br>By submitting to GigaScience, you are aware of the journal's AI-writing tools policy, and if you have declared use of such tools below, you have acknowledged this where appropriate in your manuscript and have made a summary of use and outputs available. <p><br><b>AI-assisted writing tools have been used in the preparation of this manuscript?	Yes

Powered by Editorial Manager® and ProduXion Manager® from Aries Systems Corporation

Manuscript

Click here to access/download;Manuscript;main.tex

GigaScience, 2025, 1–4

doi: xx.xxxx/xxxx
Manuscript in Preparation
Data Note

DATA NOTE

MultiMS^2: A Curated Multi-Modal, Multi-Energy Spectral Library for Metabolomics

Adriano Rutz[1,*], Mario S. P. Correia[1,*] and Nicola Zamboni[1,†]

[1]Institute for Molecular Systems Biology, ETH Zürich, Otto-Stern-Weg 3, 8093 Zürich, Switzerland

[*]Contributed equally.
[†]zamboni@imsb.biol.ethz.ch

Abstract

Background: Spectral libraries are essential for mass spectrometry-based metabolomics, enabling accurate metabolite annotation. Collision-induced dissociation (CID) dominates existing public libraries, but is rarely sufficient for structural elucidation. Electron-activated dissociation (EAD) provides complementary, radical-driven fragmentation, but remains sparsely represented. The lack of datasets spanning multiple dissociation mechanisms, energies, and ionization modes limits both analytical workflows and the development of robust machine learning models.
Findings: We present MultiMS^2, a curated metabolomics spectral library comprising 43,728 MS/MS spectra from 2,899 unique compounds. Spectra were acquired using both CID and EAD at three energies each, in positive and negative ionization modes. The dataset substantially expands publicly available EAD coverage while preserving matched acquisition conditions across energies and dissociation types.
Conclusions: By systematically combining CID and EAD across multiple energies and polarities, MultiMS^2 provides a unique resource for metabolite annotation, benchmarking, and machine learning. The library supports energy-aware and dissociation-aware analyses, enabling methodological innovation and improved generalization in computational metabolomics.

Key words: Spectral library; Collision-induced dissociation; Electron-activated dissociation; Metabolomics

Context

Metabolomics relies heavily on tandem mass spectrometry (MS^2) to characterize and annotate small molecules in biological systems. Confident metabolite annotation typically depends on comparison to reference spectral libraries. In recent years, machine learning has emerged as a central approach for automated annotation, spectrum prediction, and structure elucidation, but its success depends critically on access to large, diverse, and well-annotated training datasets.

Most existing public metabolomics libraries are dominated by CID spectra. While CID is robust and widely available, it often favors low-energy fragmentation pathways and may miss structurally informative cleavages. In contrast, EAD generates complementary radical-driven fragment ions that can enhance structural elucidation. Despite this potential, EAD spectra remain scarce in public repositories, restricting both manual interpretation and the ability of machine learning models to generalize across fragmentation mechanisms.

The accessibility and standardization of MS^2 data has substantially advanced thanks to long-standing community repositories like GNPS [1] or MassBank [2], harmonization efforts in large-scale MS/MS library curation [3, 4], and large individual initiatives [5, 6, 7]. However, these resources typically lack systematic coverage across multiple energies and dissociation mechanisms for the same set of compounds, leaving an important gap for workflows and computational models requiring broad fragmentation diversity.

To address this gap, we present MultiMS^2, a curated spectral library that systematically combines CID and EAD across three energies in both positive and negative ionization modes. Through rigorous curation and quality control, this resource aims to improve metabolite annotation and to provide a benchmark dataset for developing and evaluating machine learning methods that are robust to fragmentation physics and acquisition conditions.

Compiled on: December 17, 2025.
Draft manuscript prepared by the author.

Key Points

- Comprehensive spectral library for metabolomics spanning three energies and both polarities.
- Includes both collision-induced and electron-activated dissociation, greatly expanding EAD coverage.
- Enables improved metabolite annotation, machine learning, and method development.

Methods

Experimental

We analyzed three libraries of pure chemical standards. First, the Human Endogenous Metabolite Compound Library (1,000 standards; Selleck Chemicals, Art. No. L4500), which was pooled in sets of 10 compounds and diluted with 10% (v/v) Ethanol to a final concentration of 10 μM for injection. Second, the Mass Spectrometry Metabolite Library (MSMLS; Merck, Art. No MSMLS-1EA, Lot 2016), which was dissolved and diluted according to the manufacturer instructions with water for plates 1–5 and methanol for plates 6–7. Compounds were pooled in sets of 10 and diluted to a final concentration of 5-20 μM. Third, A library of ca. 3000 natural poducts-like compounds was obtained from NEXUS, the chemical screening facility of our Institution, prepooled in sets of 10. Compounds were diluted with 10% (v/v) Ethanol to a final concentration of 10 μM for injection.

Spectra were acquired using a SCIEX ZenoTof 7600 System coupled to an Agilent Infinity II LC stack. Direct injection (5 μL) was performed using a mobile phase made of 50:50 mixture of water:methanol (both containing 0.1% formic acid) with a flow rate of 0.2 mL/min. TOFMS data were acquired from 50 to 1500 *m/z* with an accumulation time of 50ms, declustering potential of 50 V, collision energy of 10 V, curtain gas at 45 (arbitrary units), CAD gas at 7 (arbitrary unity), ion source gas 1 and 2 at 70 psi, source temperature at 700 °C, and a spray voltage of 5500 V for positive mode and -4500 V for negative mode. Information-dependent acquisition (IDA) selected up to two ions per cycle for MS/MS, with dynamic background subtraction enabled. Zeno pulsing was applied with a threshold of 20,000 cps. Precursor ions were targeted with a mass tolerance of 50 mDa and an exclusion window of 2 s. Three collision energies were set for CID (20, 40, 60 V) and EAD, respectively (12, 16, 24 electron kinetic energy, with a current of 3500 V and 30 ms activation time). The total method duration was 0.6 min (actual acquisition time 1.06 min), with 188 estimated cycles per run.

Data processing

Raw *.wiff* data were converted to profile *.mzML* using ProteoWizard (v3.0.25182) (RRID:SCR_012056). Centroiding was performed using CentroidR (v0.0.0.9001) [8]. Spectral library was built using mzmine (v.4.7.27) (RRID:SCR_012040) and custom Python programming language programs (RRID:SCR_008394) (archived at Zenodo (RRID:SCR_004129)) [9]. Annotations include SMILES [10], InChI and InChIKeys [11], and SELFIES representations [12], along with complete instrument and acquisition metadata. Spectra are distributed in mzML and MGF formats with accompanying metadata tables.

Data validation and quality control

The spectra were inspected using a combination of automated and manual quality control procedures to ensure correct precursor assignment, spectral purity, and annotation accuracy. From the initial 148,888 candidate spectra collected, thresholds for precursor purity and spectral quality were applied uniformly across modalities, and all retained spectra passed these criteria. A minimal precursor height of 1,000 counts was required together with a minimal precursor purity of 0.9. To be retained, spectra had to be present in at least 2 modalities. The minimal number of fragments was set to 3, with at least 5% explained signals and 40% explained intensity. If multiple spectra per modality were left, only the ones with at least 40% of the maximal explained signals and 80% of the maximal explained intensity were kept. This allowed to keep multiple replicates per modality while ensuring quality. Key dataset statistics are summarized in Table 1. Representative results and validation workflows are documented in https://github.com/zamboni-lab/MultiMS2 and archived at [9].

Table 1. Key statistics of the MultiMS^2 spectral library.

Item	Quantity
Unique compounds	2899 *
Unique compound-adduct modalities	4210
Unique compound-adduct-fragmentation modalities	17170
Unique spectra	43728

* As defined by the connectivity information encoded in the first 14 characters of the corresponding InChIKey (See https://en.wikipedia.org/wiki/International_Chemical_Identifier#InChIKey).

Figure 1 shows modality overlaps using upset plots, complementing the absolute counts in Table 1 by revealing the actual extent of feature sharing; specifically, shared compound identities (Panel A) and compound–adduct pairs (Panel B). Panel A reveals strong ionization-mode specificity: the two largest intersections correspond to compounds detected exclusively in positive or negative ionization, underscoring the chemical selectivity of each mode. The fourth-largest intersection (156 compounds) includes features detected across all positive modalities except negative EAD, consistent with the known limitations of electron attachment dissociation (EAD) for anions, where low electron affinity and poor fragmentation efficiency reduce detection coverage. Ongoing methodological improvements aim to address this gap [13]. In total, 676 compounds (488 + 156 + 32) were consistently detected across all positive-mode modalities.

Spectral quality was assessed using MSBuddy [14], one of the few tools explicitly designed to account for radical-driven fragmentation in subformula assignment. This is a critical feature for evaluating EAD spectra, where unpaired electrons dominate dissociation pathways. Unlike conventional tools optimized for even-electron CID fragmentation, MSBuddy does not penalize spectra with odd-electron fragments, making it better suited for cross-modal comparison.

As shown in Figure 2, CID spectra yielded higher average molecular formula assignment probabilities compared to EAD spectra. Similarly, the fraction of fragment intensity explained by assigned subformulae was higher for CID. While these differences reflect the inherent complexity of radical-mediated fragmentation in EAD, the different information within the spectrum might help for finer structural elucidation and not particularly for formula determination. The slightly lower scores for EAD may also reflect the presence of multicharged ions, which are more prevalent in EAD spectra. Finally, on all A, B, and C panels, increasing fragmentation energy was beneficial for CID, while detrimental for EAD.

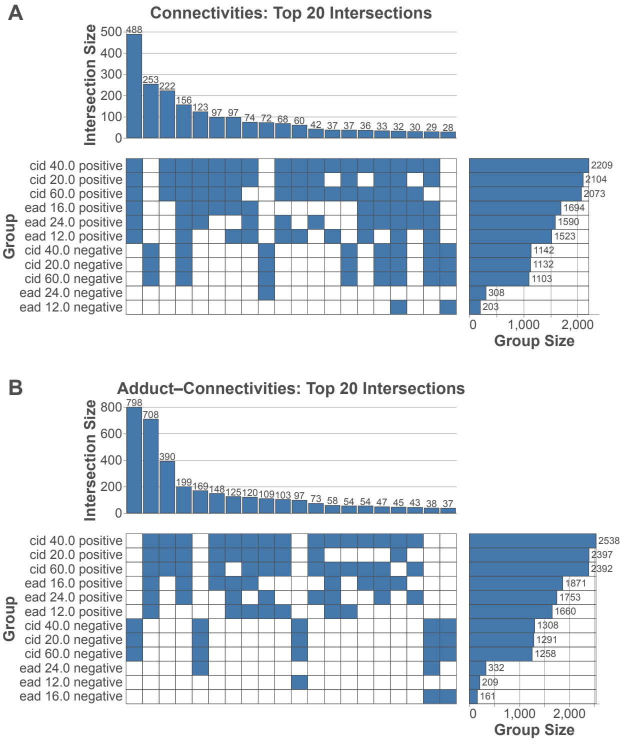

Figure 1. Overlaps between modalities
Total group sizes are shown on the right, and intersection sizes at the top. Only the top 20 intersections are displayed.
Panel A: Overlap of compounds fragmented across modalities (*e.g.*, 676 compounds (488 + 156 + 32) in all positive modalities).
Panel B: Overlap of compound-adduct pairs, considering both molecular ion and adduct type. Trends mirror Panel A, except no adduct types are shared between negative and positive ionizations.

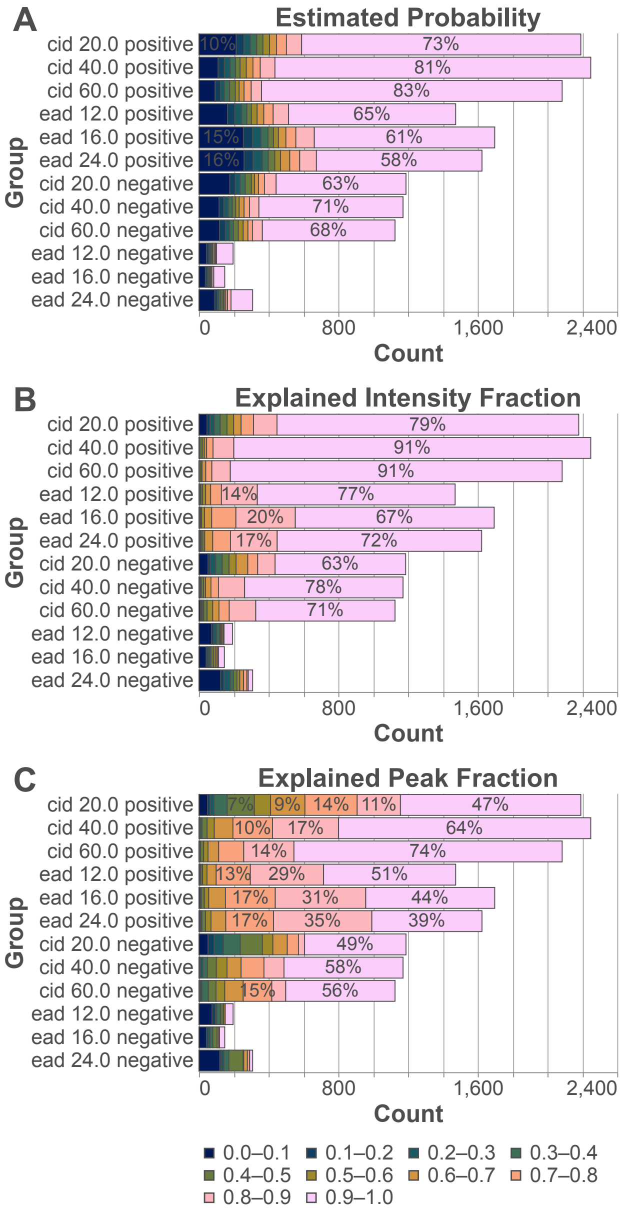

Figure 2. Spectral quality metrics from BUDDY
Some of the metrics calculated by BUDDY were used as proxies to assess spectral quality.
Panel A: Estimated probability of the assigned molecular formula. Overall, calculated probabilities were high (around 80% above 0.9 for CID positive). Probabilities increased with higher CID energy but decreased for EAD. Probabilities were lower in negative mode.
Panel B: Fraction of total MS2 intensity explained by subformulae. Similar to Panel A, the proportion of spectra considered high-quality by this metric was generally high.
Panel C: Fraction of total fragment count explained by subformulae. This complements Panel B, since a single very intense ion could otherwise bias the interpretation.

Re-use potential

MultiMS^2 significantly enhances metabolite annotation in both untargeted and targeted metabolomics by offering systematic, multi-dimensional coverage of dissociation mechanisms, collision energies, and ionization polarities. This structured design makes it uniquely suited for training and evaluating machine learning models, particularly for tasks such as:

- **Fragmentation prediction**: Modeling how molecules break under varying conditions.
- **Energy-aware modeling**: Incorporating collision energy as a continuous variable to improve spectral simulation.
- **Cross-dissociation transfer learning**: Leveraging knowledge from one fragmentation technique to improve performance on another.

The dataset also serves as a benchmark for model robustness, enabling direct comparison of algorithm performance across different fragmentation techniques.

Beyond machine learning, MultiMS^2 can be integrated into existing spectral matching platforms, enhancing annotation confidence through multi-modal spectral libraries. It supports workflows in environmental screening, clinical metabolomics, and systems biology.

Availability of source code and requirements

- Project name: MultiMS^2
- Project repository: `https://github.com/zamboni-lab/MultiMS2`
- Operating system(s): Platform independent (Docker container provided)
- Programming language:
  - Python Programming Language (RRID:SCR_008394)
  - R Project for Statistical Computing (RRID:SCR_001905)
  - Bash (RRID:SCR_021268)
- Other requirements:
  - Docker Desktop (RRID:SCR_016445)
  - ProteoWizard (RRID:SCR_012056)
  - mzmine (RRID:SCR_012040)
  - uv (`https://docs.astral.sh/uv/`)
- License: MIT License

- Any restrictions to use by non-academics: None

Data availability

The data sets supporting the results of this article are available in both Zenodo [15] and MassIVE repositories [16] under permissive CC0 1.0 Universal License.

Declarations

List of abbreviations

- **CID**: Collision-Induced Dissociation
- **EAD**: Electron-Activated Dissociation
- **InChI(Key)**: International Chemical Identifier (Key)
- **MassIVE**: Mass Spectrometry Interactive Virtual Environment
- **SELFIES**: Self-Referencing Embedded Strings
- **SMILES**: Simplified Molecular Input Line Entry System

Ethical Approval

Not applicable

Consent for publication

Not applicable

Competing Interests

The authors declare that they have no competing interests.

Funding

This work was supported by a grant from the Swiss National Science Foundation (project MetabolinkAI, #10002786), and a grant from the Strategic Focal Area Personalized Health and Related Technologies (PHRT) of the ETH Domain (#603).

Author's Contributions

Conceptualization: A.R. and N.Z. Data curation: A.R. Formal analysis: A.R. Funding acquisition: N.Z. Investigation: M.S.P.C. Methodology: A.R. and N.Z. Project administration: N.Z. Resources: N.Z. Software: A.R. Supervision: N.Z. Validation: A.R. and M.S.P.C. Visualization: A.R. Writing-original draft: A.R. Writing-review and editing: A.R. and N.Z.

Acknowledgements

The authors used AI-assisted language models solely for language editing and clarity improvement. No scientific content, data analysis, or conclusions were generated by the tool. All outputs were critically reviewed by the authors.

References

1. Wang M, Carver JJ, Phelan VV, Sanchez LM, Garg N, Peng Y, et al. Sharing and community curation of mass spectrometry data with Global Natural Products Social Molecular Networking. Nature Biotechnology 2016 Aug;34(8):828–837. http://dx.doi.org/10.1038/nbt.3597.
2. Neumann S, Meier R, Wenk M, Elapavalore A, Nishioka T, Schulze T, et al. MassBank: an open and FAIR mass spectral data resource. Nucleic Acids Research 2025 Nov;http://dx.doi.org/10.1093/nar/gkaf1193.
3. de Jonge NF, Hecht H, Strobel M, Wang M, van der Hooft JJJ, Huber F. Reproducible MS/MS library cleaning pipeline in matchms. Journal of Cheminformatics 2024 Jul;16(1). http://dx.doi.org/10.1186/s13321-024-00878-1.
4. Gupta V, Qiang H, Chung HH, Herbst E, Skinnider M. Comprehensive curation and harmonization of small molecule MS/MS libraries in Spectraverse 2025 Oct;http://dx.doi.org/10.26434/chemrxiv-2025-4pzxn.
5. Kong F, Keshet U, Shen T, Rodriguez E, Fiehn O. LibGen: Generating High Quality Spectral Libraries of Natural Products for EAD-, UVPD-, and HCD-High Resolution Mass Spectrometers. Analytical Chemistry 2023 Nov;95(46):16810–16818. http://dx.doi.org/10.1021/acs.analchem.3c02263.
6. Brungs C, Schmid R, Heuckeroth S, Mazumdar A, Drexler M, Šácha P, et al. MSnLib: efficient generation of open multistage fragmentation mass spectral libraries. Nature Methods 2025 Sep;22(10):2028–2031. http://dx.doi.org/10.1038/s41592-025-02813-0.
7. Singh Y, Norris PC, Maharjan S, Gillespie J, Ferrante C, Ibrahim Z, et al. CIeaD: A Complementary CID and EAD Mass Spectral Library for Phytochemicals. Journal of the American Society for Mass Spectrometry 2025 Dec;http://dx.doi.org/10.1021/jasms.5c00329.
8. Rutz A, Rainer J, CentroidR: Repository to centroid profile spectra. Zenodo; 2025. https://zenodo.org/doi/10.5281/zenodo.17250307.
9. Rutz A, Povoa Correia MS, Zamboni N, MultiMS2 spectral library - MGF and processing workflow. Zenodo; 2025. https://zenodo.org/doi/10.5281/zenodo.17417089.
10. Weininger D. SMILES, a chemical language and information system. 1. Introduction to methodology and encoding rules. Journal of Chemical Information and Computer Sciences 1988 Feb;28(1):31–36. http://dx.doi.org/10.1021/ci00057a005.
11. Heller SR, McNaught A, Pletnev I, Stein S, Tchekhovskoi D. InChI, the IUPAC International Chemical Identifier. Journal of Cheminformatics 2015 May;7(1). http://dx.doi.org/10.1186/s13321-015-0068-4.
12. Krenn M, Häse F, Nigam A, Friederich P, Aspuru-Guzik A. Self-referencing embedded strings (SELFIES): A 100string representation. Machine Learning: Science and Technology 2020 Oct;1(4):045024. http://dx.doi.org/10.1088/2632-2153/aba947.
13. Karasawa K, Duchoslav E, Baba T. Fast Electron Detachment Dissociation of Oligonucleotides in Electron-Nitrogen Plasma Stored in Magneto Radio-Frequency Ion Traps. Analytical Chemistry 2022 Oct;94(44):15510–15517. http://dx.doi.org/10.1021/acs.analchem.2c04027.
14. Xing S, Shen S, Xu B, Li X, Huan T. BUDDY: molecular formula discovery via bottom-up MS/MS interrogation. Nature Methods 2023 Apr;20(6):881–890. http://dx.doi.org/10.1038/s41592-023-01850-x.
15. Povoa Correia MS, Rutz A, Zamboni N, MultiMS2 spectral library - mzml positive and negative. Zenodo; 2025. https://zenodo.org/doi/10.5281/zenodo.17250693.
16. Zamboni N, MassIVE MSV000099369 - GNPS - MultiMS2 spectral library. MassIVE; 2025. https://massive.ucsd.edu/ProteoSAFe/dataset.jsp?accession=MSV000099369.
