# Supplementary material for: MultiMS2: A curated multi-modal, multi-energy spectral library for metabolomics
Source: Gigascience. 2026 Jun 10;15:giag069. doi: 10.1093/gigascience/giag069 (PMC13312951; doi:10.1093/gigascience/giag069)

GigaScience

MultiMS2: A Curated Multi-Modal, Multi-Energy Spectral Library for Metabolomics

--Manuscript Draft--

**Manuscript Number:**	GIGA-D-25-00518R1
**Full Title:**	MultiMS2: A Curated Multi-Modal, Multi-Energy Spectral Library for Metabolomics
**Article Type:**	Data Note
**Funding Information:**	Schweizerischer Nationalfonds zur Förderung der Wissenschaftlichen Forschung (10002786) — Prof. Nicola Zamboni<br>Eidgenössische Technische Hochschule Zürich (23-2 ETH-037) — Prof. Nicola Zamboni
**Abstract:**	Background:<br>Spectral libraries are essential for mass spectrometry-based metabolomics, enabling accurate metabolite annotation. Collision-induced dissociation (CID) dominates existing public libraries, but is rarely sufficient for structural elucidation. Electron-activated dissociation (EAD) provides complementary, radical-driven fragmentation, but remains sparsely represented. The lack of datasets spanning multiple dissociation mechanisms, energies, and ionization modes limits both analytical workflows and the development of robust machine learning models.<br>Findings:<br>We present MultiMS2, a curated metabolomics spectral library comprising 43,728 MS/MS spectra from 2,899 unique compounds. Spectra were acquired using both CID and EAD at three energies each, in positive and negative ionization modes. The dataset substantially expands publicly available EAD coverage while preserving matched acquisition conditions across energies and dissociation types.<br>Conclusions:<br>By systematically combining CID and EAD across multiple energies and polarities, MultiMS2 provides a unique resource for metabolite annotation, benchmarking, and machine learning. The library supports energy-aware and dissociation-aware analyses, enabling methodological innovation and improved generalization in computational metabolomics.
**Corresponding Author:**	Nicola Zamboni<br>ETH Zürich D-BIOL: Eidgenossische Technische Hochschule Zurich Departement Biologie<br>Zurich, SWITZERLAND
**Corresponding Author Secondary Information:**	
**Corresponding Author's Institution:**	ETH Zürich D-BIOL: Eidgenossische Technische Hochschule Zurich Departement Biologie
**Corresponding Author's Secondary Institution:**	
**First Author:**	Adriano Rutz
**First Author Secondary Information:**	
**Order of Authors:**	Adriano Rutz
	Mario Sergio Povoa Correia
	Nicola Zamboni
**Order of Authors Secondary Information:**	
**Response to Reviewers:**	## Reviewer #1<br><br>> The manuscript has described MultiMS2: ACuratedMulti-Modal,Multi-Energy Spectral Library for Metabolomics. However, there are several key aspects that need to be

Powered by Editorial Manager® and ProduXion Manager® from Aries Systems Corporation

clarified and further verified to enhance the scientific rigor and impact of the research. I recommend accepting the paper after minor revisions.

1. EAD energies are 12, 16, 24 eV, what is the consideration for the large span between the second and third energies?

We were hoping to capture possibly diverse dissociation events.
12 and 16 eV cover the range that has been previously reported to be optimal for lipids, bile acids, steroids and others.
Rather staying in the same range, we hypothesized tha higher energies might help to increase the yield of different types of fragments, as we saw for e.g. lipids.
Given the chemodiversity of our collection.
In addition, high kinetic energy are necessary for the activation of anions in negative mode ionization (see next point).
Hence, 24 was more appropriate to complement 12 and 16 eV.

2. The quality of EAD data in negative ion mode is far worse than that of other modes, and whether it is necessary to discuss it separately.

The lower number of fragments obtained by EAD in negative mode is not an indicator of poor quality, but the direct consequence of the electrostatic repulsion between the anionic compounds and electrons.
The lower coverage in negative mode is an expected, physical limitation of electron activation methods.
This is well-established in the EAD/ECD literature.

3. Since the results in figure 2 show that the interpretability of EAD data is worse, how to achieve the prediction based on EAD data mentioned by the author later.

We respectfully disagree with the Reviewer's claim that Figure 2 shows "interpretability of EAD data is worse".
We assessed formula assignment as a first-pass quality metric.
This refers to the composition of fragments and de novo formula prediction from MS2.
In contrast, it reveals nothing about structural elucidation capabilities, which may also well be superior for EAD.
More in general, we would like to stress that EAD and CID should no be antagonized.
The fundamental question is how the two modalities synergize.
Given the broad availability of CID spectra and CID-specific AI tools, we think that also in the future CID will be used in a first pass to infer the molecular formula and obtain a shortlist of putative candidates.
EAD could then be adopted in a second pass to rerank shortlisted compounds, exploiting EAD-specific fragments.

4. The necessity of using EAD-MS/MS has been repeatedly emphasized in the introduction and abstract, but the unique advantages and application scenarios of EAD-MS/MS are not reflected in the results.

We agree with the Reviewer and acknowledge this gap, but this is what a "Gigascience Data Note" is about: providing non-existing, high-quality data to the community to enable future studies that can systematically evaluate EAD advantages
We kept the comments on the advantages of EAD-MS/MS because they refer to previous studies that focused on specific classes.
We don't make any specific claims about this dataset.

5. "Multidimensional Mass Spectral Similarity Algorithm: Discriminate Disaccharide and Flavonoid Isomers Coupled with Online Energy-Resolved Acquisition and Electron Activation Dissociation Anal. Chem. 2025, 97, 26781−26792; Energy-resolved in-source dissociation spectra by tunable pulsed arc plasma ionization for the differentiation of structural isomers Chinese Chemical Letters https://doi.org/10.1016/j.cclet.2025.112060" should be cited.

We don't understand where this work should be cited and for what reason.
The first paper indeed used EAD on either disaccharides or flavonoids, but the it focuses on a modified spectrum similarity metrics. They don't make any spectrum

available (other than in a low res PDF). This seems unrelated for the generic used of EAD, which doesn't justify a citation, for the same reason we don't cite dozens of other EAD papers.

The second paper is even less related: in-source fragmentation by plasma? it's from the same lab as above, but we struggle to see any link to our own contribution. There is no EAD, no spectral library. Please provide more guidance on where and why these two papers should be cited.

Reviewer #2

> Overall the MultiMs2 address a gap in EAD spectral library. The library is only shared as raw data files which limits its reuse as reference library. The major concern is negative mode datasets which is far poor coverage than positive mode datasets. This is contrary to endogenous metabolomics literature where negative mode outperforms positive mode. This suggests some major drawback in data acquisition or analysis methodology.

1. Is there any stat or reference available about breakdown of CID vs EAD spectral data in public libraries?

The short answer is no. It's hard to systematically search for EAD in repositories such as MoNA, GNPS, etc. because EAD is not reported in a systemstic way in the records. There is a lack of standardization.
We have to rely on primary literature, and the only sizeable EAD library appears to be the one published by the Fiehn lab in their LibGen publication (and more recently CIeaD, but it lacks structural identifiers).
This is symptomatic for the difficulty of obtaining solid stats.
This context strengthens the rationale for MultiMS2 as a resource.

2. METLIN and some other spectral libraries are exception to this which can be acknowledged

We rephrased to "these resources typically lack systematic coverage across dissociation mechanisms and multiple energies" to stress that the main limitation is multiple dissociatin mechanisms.
We are not aware that METLIN or other libraries provide multi-energy EAD spectra for metabolites, but this might be due to the difficulty obtaining the information (see #1).
If the reviewwer is aware of any accessible and citable source, we would be glad to correct the manuscript.

3. MedChem express website suggests Human Endogenous Metabolite Compound Library is consists of pre-disolved solutions of 825 metabolites
"Human Endogenous Metabolite Compound Library (1,000 standards; Selleck Chemicals, Art. No. L4500), which was pooled in sets of 10 compounds and diluted with 10% (v/v) Ethanol to a final concentration of 10 µM for injection."

We confirm the correctness of the statement.
At the time of purchase of L4500, we received 1013 compounds distributed across 13 96 WPs, all with associated metadata.
It seems that in the meantime, the number was reduced to 840.
Importantly, this does not affect any of our data, analyses, or conclusions.

4. The practical challenge in preparation of these compounds is solubility in single solvent or solvent mixtures (10% ethanol may not be accurate). Can authors discuss or cite how they addressed such issues? Also, the mixture of 10 compounds is based on non-overlapping precursor masses?

We thank the reviewer for raising this practical point.
We have added a clarifying note to the Methods section.
Compounds were used as supplied by the manufacturer (pre-dissolved) or prepared following manufacturer protocols.
Compound pooling was designed to maximize throughput; pools were assembled to

minimize precursor mass overlap, and any remaining conflicts were resolved during data processing, consistent with standard practice in large-scale spectral library acquisition workflows.

5. For direct injection buffered mobile phase with formic acid may not be adequate for negative ionization mode. This may contribute to the poor performance of negative mode ionization datasets. Authors strongly recommended using ammonium hydroxide, or other basic modifiers for negative mode ionization.

We thank the reviewer for this constructive observation.
We have added a note to the manuscript acknowledging that the use of formic acid as a mobile phase modifier, while standard for positive mode ESI, is suboptimal for negative mode ionization.
Future work and library and updates will explore the use of alkaline modifiers such as ammonium hydroxide to improve negative mode coverage.

6. What is rational for selecting 3 CEs?

We chose 3 energies for practical reasons.
We had to acquire all MS2 spectra of several compounds incl. multiple adducts during the apex of the elution peak.
Because of limitation in the acquisition software, we had to use DDA, which lead to the fragmentation of background ions.
The DDA engine has a powerful dynamic background substraction that prevents to select constant signals, but it only works in combination with a chromatographic separation.
Since we used flow injection, the dynamic background substraction wasn't able to distinguish analytes of interest from background, and had to be deactivated.
We partially mitigated the problem with a static exclusion list, but still had to take into account fragmenting unwanted precursors before DDA selected the adducts of interest.
The selection of three collision energies follows the approach established by other spectral libraries, which demonstrated that coverage of low, medium, and high fragmentation regimes captures complementary structural information without excessive redundancy.
Once the number was set, we picked 3 energy values to span the whole range that we typically use.
Note that the baseline kinetic energy in TOFMS mode is 10 eV.

7. What is biochemical pathway and/or chemical class specific breakdown of the presented compounds?

This is a fair and valuable question.
We have generated a chemical class and pathway annotation map using TMAP, which has been deposited in the repository alongside the spectral data.
A reference to this visualization has been added to the manuscript, allowing users to rapidly assess the chemical space covered by MultiMS2.

8. Figure 1 is clearly presented and only highlights poor coverage for negative mode datasets. This contradicts with literature and general observation about endogenous metabolites where negative mode outperforms positive mode. Again, authors should revisit their data acquisition and processing strategy to address this concern.

We respectfully note that the composition of the library reflects the ionization properties of the commercially available standards used, which are enriched in compounds that preferentially ionize in positive mode.
This is consistent with observations reported for comparable spectral libraries (e.g., Brungs et al., MSnlib).
The observed positive-mode enrichment is therefore a characteristic of the compound collection, not a methodological failure.
Please note that the low numbers reported for EAD in negative mode are caused by well-known physical limitations of ExD, not by acquisition or processing.

9. Reuse and data availability: raw data is provided as files. This is appreciated,

	however, for reuse of the library for non-computational chemists this will cumbersome task to extract MS2 data and REUSE it as a reference library. It is recommended that library to be part of MassBank or MONA repositories in sharable MSP or MGF formats.<br><br>All spectra have been deposited in GNPS in MGF format, as described in the Data Availability section, and are additionally available on Zenodo.<br>Raw files are provided for full transparency and to enable reprocessing.<br>We believe this addresses the reviewer's concern.<br><br>10. Several key references of other spectral libraries are missing.<br><br>We thank the reviewer for this suggestion and have carefully reviewed the reference list.<br>We have incorporated additional citations to key spectral resources throughout the manuscript where most relevant.
**Additional Information:**	
**Question**	**Response**
Are you submitting this manuscript to a special series or article collection?	No
**Experimental design and statistics**<br><br>Full details of the experimental design and statistical methods used should be given in the Methods section, as detailed in our Minimum Standards Reporting Checklist. Information essential to interpreting the data presented should be made available in the figure legends.<br><br>Have you included all the information requested in your manuscript?	Yes
**Resources**<br><br>A description of all resources used, including antibodies, cell lines, animals and software tools, with enough information to allow them to be uniquely identified, should be included in the Methods section. Authors are strongly encouraged to cite Research Resource Identifiers (RRIDs) for antibodies, model organisms and tools, where possible.<br><br>Have you included the information requested as detailed in our Minimum Standards Reporting Checklist?	Yes

Powered by Editorial Manager® and ProduXion Manager® from Aries Systems Corporation

**Availability of data and materials**<br><br>All datasets and code on which the conclusions of the paper rely must be either included in your submission or deposited in publicly available repositories (where available and ethically appropriate), referencing such data using a unique identifier in the references and in the "Availability of Data and Materials" section of your manuscript.<br><br>Have you have met the above requirement as detailed in our Minimum Standards Reporting Checklist?	Yes
GigaScience has policies and guidelines in place for the use of generative AI-writing tools such as ChatGPT. If you have used such writing tools to assist with writing the manuscript this must be declared and cited in the text. Authors should not list AI-writing tools and other AI-assisted technologies as an author or co-author and should acknowledge that they are fully responsible for text generated or refined by AI-writing tools.<p><br><br>A summary of use (particularly in the introduction or among methods) needs to be included at the end of the paper, and the outputs should also be included as a supplementary file hosted in GigaDB or other open repositories. Please read our guidelines for more information. <p><br><br>By submitting to GigaScience, you are aware of the journal's AI-writing tools policy, and if you have declared use of such tools below, you have acknowledged this where appropriate in your manuscript and have made a summary of use and outputs available. <p><br><b>AI-assisted writing tools have been used in the preparation of this manuscript?	Yes

Powered by Editorial Manager® and ProduXion Manager® from Aries Systems Corporation

Manuscript Click here to access/download;Manuscript;main.pdf

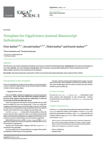

GigaScience, 2025, 1–6

doi: xx.xxxx/xxxx
Manuscript in Preparation
Data Note

DATA NOTE

MultiMS^2: A Curated Multi-Modal, Multi-Energy Spectral Library for Metabolomics

Adriano Rutz[1,*], Mario S. P. Correia[1,*] and Nicola Zamboni[1,†]

[1]Institute for Molecular Systems Biology, ETH Zürich, Otto-Stern-Weg 3, 8093 Zürich, Switzerland

[*]Contributed equally.
[†]zamboni@imsb.biol.ethz.ch

Abstract

Background: Spectral libraries are essential for mass spectrometry-based metabolomics, enabling accurate metabolite annotation. Collision-induced dissociation (CID) dominates existing public libraries, but is rarely sufficient for structural elucidation. Electron-activated dissociation (EAD) provides complementary, radical-driven fragmentation, but remains sparsely represented. The lack of datasets spanning multiple dissociation mechanisms, energies, and ionization modes limits both analytical workflows and the development of robust machine learning models.
Findings: We present MultiMS^2, a curated metabolomics spectral library comprising 43,728 MS/MS spectra from 2,899 unique compounds. Spectra were acquired using both CID and EAD at three energies each, in positive and negative ionization modes. The dataset substantially expands publicly available EAD coverage while preserving matched acquisition conditions across energies and dissociation types.
Conclusions: By systematically combining CID and EAD across multiple energies and polarities, MultiMS^2 provides a unique resource for metabolite annotation, benchmarking, and machine learning. The library supports energy-aware and dissociation-aware analyzes, enabling methodological innovation and improved generalization in computational metabolomics.

Key words: Spectral library; Collision-induced dissociation; Electron-activated dissociation; Metabolomics

Context

Metabolomics relies heavily on tandem mass spectrometry (MS^2) to characterize and annotate small molecules in biological systems. Confident metabolite annotation typically depends on comparison to reference spectral libraries. In recent years, machine learning has emerged as a central approach for automated annotation, spectrum prediction, and structure elucidation, but its success depends critically on access to large, diverse, and well-annotated training datasets.

Most existing public metabolomics libraries are dominated by CID spectra. While CID is robust and widely available, it often favors low-energy fragmentation pathways and may miss structurally informative cleavages. In contrast, EAD generates complementary radical-driven fragment ions that can enhance structural elucidation. Despite this potential, EAD spectra remain scarce in public repositories, restricting both manual interpretation and the ability of machine learning models to generalize across fragmentation mechanisms.

The accessibility and standardization of MS^2 data has substantially advanced thanks to long-standing community repositories like GNPS [1] or MassBank [2] and its north american version (RRID:SCR_015536), harmonization efforts in large-scale MS/MS library curation [3, 4], and large individual initiatives [5, 6, 7]. However, these resources typically lack systematic coverage across dissociation mechanisms and multiple energies for the same set of compounds, leaving an important gap for workflows and computational models requiring broad fragmentation diversity. Other more comprehensive libraries such as METLIN [8] or NIST (RRID:SCR_014668) can be mentioned, but these are not openly accessible.

To address this gap, we present MultiMS^2, a curated spectral library that systematically combines CID and EAD across three energies in both positive and negative ionization modes. Through rigorous curation and quality control, this resource aims to improve

Compiled on: April 22, 2026.
Draft manuscript prepared by the author.

Key Points

- Comprehensive spectral library for metabolomics spanning three energies and both polarities.
- Includes both collision-induced and electron-activated dissociation, greatly expanding EAD coverage.
- Enables improved metabolite annotation, machine learning, and method development.

metabolite annotation and to provide a benchmark dataset for developing and evaluating machine learning methods that are robust to fragmentation physics and acquisition conditions.

Methods

Experimental

We analyzed three libraries of pure chemical standards. First, the Human Endogenous Metabolite Compound Library (ca. 1,000 standards; Selleck Chemicals, Art. No. L4500), which was pooled in sets of 10 compounds and diluted with 10% (v/v) ethanol to a final concentration of 10 μM for injection. Second, the Mass Spectrometry Metabolite Library (MSMLS; Merck, Art. No MSMLS-1EA, Lot 2016), which was dissolved and diluted according to the manufacturer instructions (water for plates 1–5, methanol for plates 6–7). Compounds were pooled in sets of 10 and diluted to a final concentration of 5-20 μM. Third, a library of ca. 3000 natural products-like compounds was obtained from NEXUS, the chemical screening facility of our Institution, pre-pooled in sets of 10. Compounds were diluted with 10% (v/v) ethanol to a final concentration of 10 μM. Overall, compounds were used as supplied by the manufacturer (pre-dissolved) or prepared following manufacturer protocols. Compound pooling was designed to maximize throughput; pools were assembled to minimize precursor mass overlap, and any remaining conflicts were resolved during data processing, consistent with standard practice in large-scale spectral library acquisition workflows.

Spectra were acquired using a SCIEX ZenoTof 7600 System coupled to an Agilent Infinity II LC stack. Direct injection (5 μL) was performed using a mobile phase made of 50:50 mixture of water:methanol (both containing 0.1% formic acid) with a flow rate of 0.2 mL/min. While standard for positive mode ESI, formic acid is suboptimal for negative mode ionization. Future work and library and updates will explore the use of alkaline modifiers such as ammonium hydroxide to improve negative mode coverage. TOFMS data were acquired from 50 to 1500 *m/z* with an accumulation time of 50ms, declustering potential of 50 V, collision energy of 10 V, curtain gas at 45 (arbitrary units), CAD gas at 7 (arbitrary unity), ion source gas 1 and 2 at 70 psi, source temperature at 700 °C, and a spray voltage of 5500 V for positive mode and -4500 V for negative mode. Information-dependent acquisition (IDA) selected up to two ions per cycle for MS/MS, with dynamic background subtraction enabled. Zeno pulsing was applied with a threshold of 20,000 cps. Precursor ions were targeted with a mass tolerance of 50 mDa and an exclusion window of 2 s. Three collision energies were set for CID (20, 40, 60 V) and EAD, respectively (12, 16, 24 electron kinetic energy, with a current of 3500 V and 30 ms activation time). The total method duration was 0.6 min (actual acquisition time 1.06 min), with 188 estimated cycles per run.

Data processing

Raw *.wiff* data were converted to profile *.mzML* using ProteoWizard (v3.0.25182) (RRID:SCR_012056). Centroiding was performed using CentroidR (v0.0.0.9001) [9]. Spectral library was built using mzmine (v.4.7.27) (RRID:SCR_012040) and custom Python programs (RRID:SCR_008394) (archived at Zenodo (RRID:SCR_004129)) [10]. Annotations include SMILES [11], InChI and InChIKeys [12], and SELFIES representations [13], along with complete instrument and acquisition metadata. Spectra are distributed in mzML and MGF formats with accompanying metadata tables.

Table 1. Key statistics of the MultiMS^2 spectral library.

Item	Quantity
Unique compounds	2899 *
Unique compound-adduct modalities	4210
Unique compound-adduct-fragmentation modalities	17170
Unique spectra	43728

* As defined by the connectivity information encoded in the first 14 characters of the corresponding InChIKey (See [15]).

Data validation and quality control

The spectra were inspected using a combination of automated and manual quality control procedures to ensure correct precursor assignment, spectral purity, and annotation accuracy. From the initial 148,888 candidate spectra collected, thresholds for precursor purity and spectral quality were applied uniformly across modalities, and all retained spectra passed these criteria. A minimal precursor height of 1,000 counts was required together with a minimal precursor purity of 0.9. To be retained, spectra had to be present in at least 2 modalities. The minimal number of fragments was set to 3, with at least 5% explained signals and 40% explained intensity. If multiple spectra per modality were left, only the ones with at least 40% of the maximal explained signals and 80% of the maximal explained intensity were kept. This allowed to keep multiple replicates per modality while ensuring quality. Key dataset statistics are summarized in Table 1. Representative results and validation workflows are documented in [14] and archived at [10].

Figure 1 shows modality overlaps using upset plots, complementing the absolute counts in Table 1 by revealing the actual extent of feature sharing; specifically, shared compound identities (Panel A) and compound–adduct pairs (Panel B). Panel A reveals strong ionization-mode specificity: the two largest intersections correspond to compounds detected exclusively in positive or negative ionization, underscoring the chemical selectivity of each mode. The fourth-largest intersection (156 compounds) includes features detected across all positive modalities except negative EAD, consistent with the known limitations of electron attachment dissociation (EAD) for anions, where low electron affinity and poor fragmentation efficiency reduce detection coverage. Ongoing methodological improvements aim to address this gap [16]. In total, 676 compounds (488 + 156 + 32) were consistently detected across all positive-mode modalities.

Spectral quality was assessed using MSBuddy [17], one of the few tools explicitly designed to account for radical-driven fragmentation in subformula assignment. This is a critical feature for evaluating EAD spectra, where unpaired electrons dominate dissociation pathways. Unlike conventional tools optimized for even-electron CID fragmentation, MSBuddy does not penalize spectra with odd-electron fragments, making it better suited for cross-modal com-

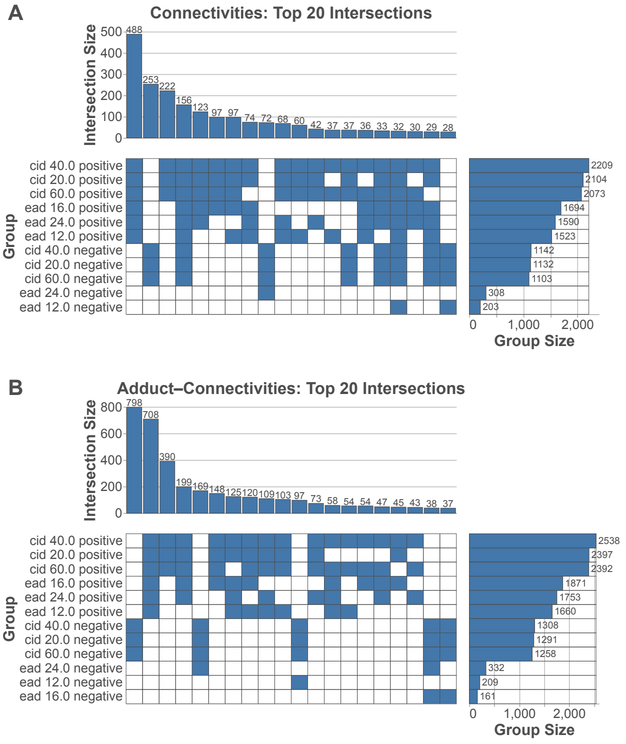

Figure 1. Overlaps between modalities
Total group sizes are shown on the right, and intersection sizes at the top. Only the top 20 intersections are displayed.
Panel A: Overlap of compounds fragmented across modalities (*e.g.*, 676 compounds (488 + 156 + 32) in all positive modalities).
Panel B: Overlap of compound-adduct pairs, considering both molecular ion and adduct type. Trends mirror Panel A, except no adduct types are shared between negative and positive ionizations.

parison.

As shown in Figure 2, CID spectra yielded higher average molecular formula assignment probabilities compared to EAD spectra. Similarly, the fraction of fragment intensity explained by assigned subformulae was higher for CID. While these differences reflect the inherent complexity of radical-mediated fragmentation in EAD, the different information within the spectrum might help for finer structural elucidation and not particularly for formula determination. The slightly lower scores for EAD may also reflect the presence of multicharged ions, which are more prevalent in EAD spectra. Finally, on all A, B, and C panels, increasing fragmentation energy was beneficial for CID, while detrimental for EAD.

To visualize the chemical diversity of the MultiMS^2 library, we embedded all compounds using MinHash fingerprints (MAP4) [18] and constructed a Tree MAP (TMAP) [19] (Figure 3). Each node represents a unique compound; proximity reflects structural similarity. The resulting map was annotated with six orthogonal metadata layers to assess how acquisition properties and chemical classifications distribute across chemical space. Panel A shows the proportion of spectra obtained in CID-only, EAD-only or in both modalities. Similarly, ionization mode (Panel B) and adduct type (Panel C) show a good coverage of different modalities per compound. Panel D shows the overlap of MultiMS^2 entries with all openly accessible spectral libraries, with some clusters only present in MultiMS^2. Panels E and F reveal that ChEBI chemical classes (computed using [20]) and NPClassifier (NPC) biosynthetic pathway assignments [21] are also diverse. Taken together, the TMAP visualization demonstrates that MultiMS^2 provides broad and structurally diverse coverage, spanning multiple compound classes and biosynthetic families, while maintaining balanced representation across the acquisition conditions central to this library.

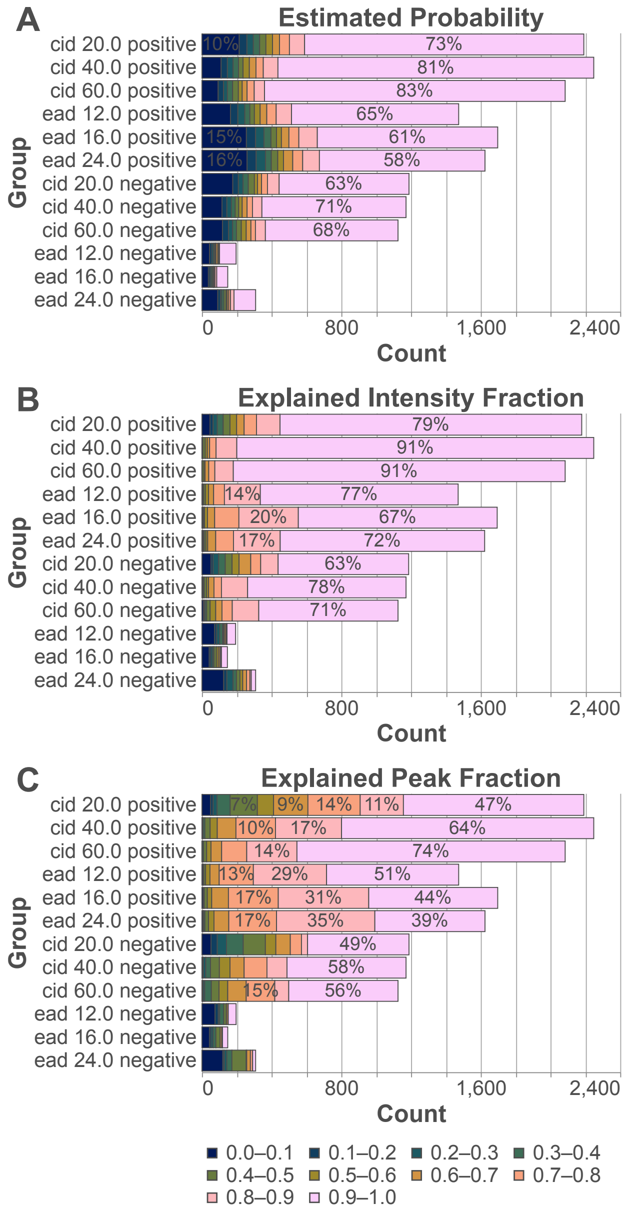

Figure 2. Spectral quality metrics from BUDDY
Some of the metrics calculated by BUDDY were used as proxies to assess spectral quality.
Panel A: Estimated probability of the assigned molecular formula. Overall, calculated probabilities were high (around 80% above 0.9 for CID positive). Probabilities increased with higher CID energy but decreased for EAD. Probabilities were lower in negative mode.
Panel B: Fraction of total MS2 intensity explained by subformulae. Similar to Panel A, the proportion of spectra considered high-quality by this metric was generally high.
Panel C: Fraction of total fragment count explained by subformulae. This complements Panel B, since a single very intense ion could otherwise bias the interpretation.

Re-use potential

MultiMS^2 significantly enhances metabolite annotation in both untargeted and targeted metabolomics by offering systematic, multidimensional coverage of dissociation mechanisms, collision energies, and ionization polarities. This structured design makes it uniquely suited for training and evaluating machine learning models, particularly for tasks such as:

- **Fragmentation prediction**: Modeling how molecules break under varying conditions.

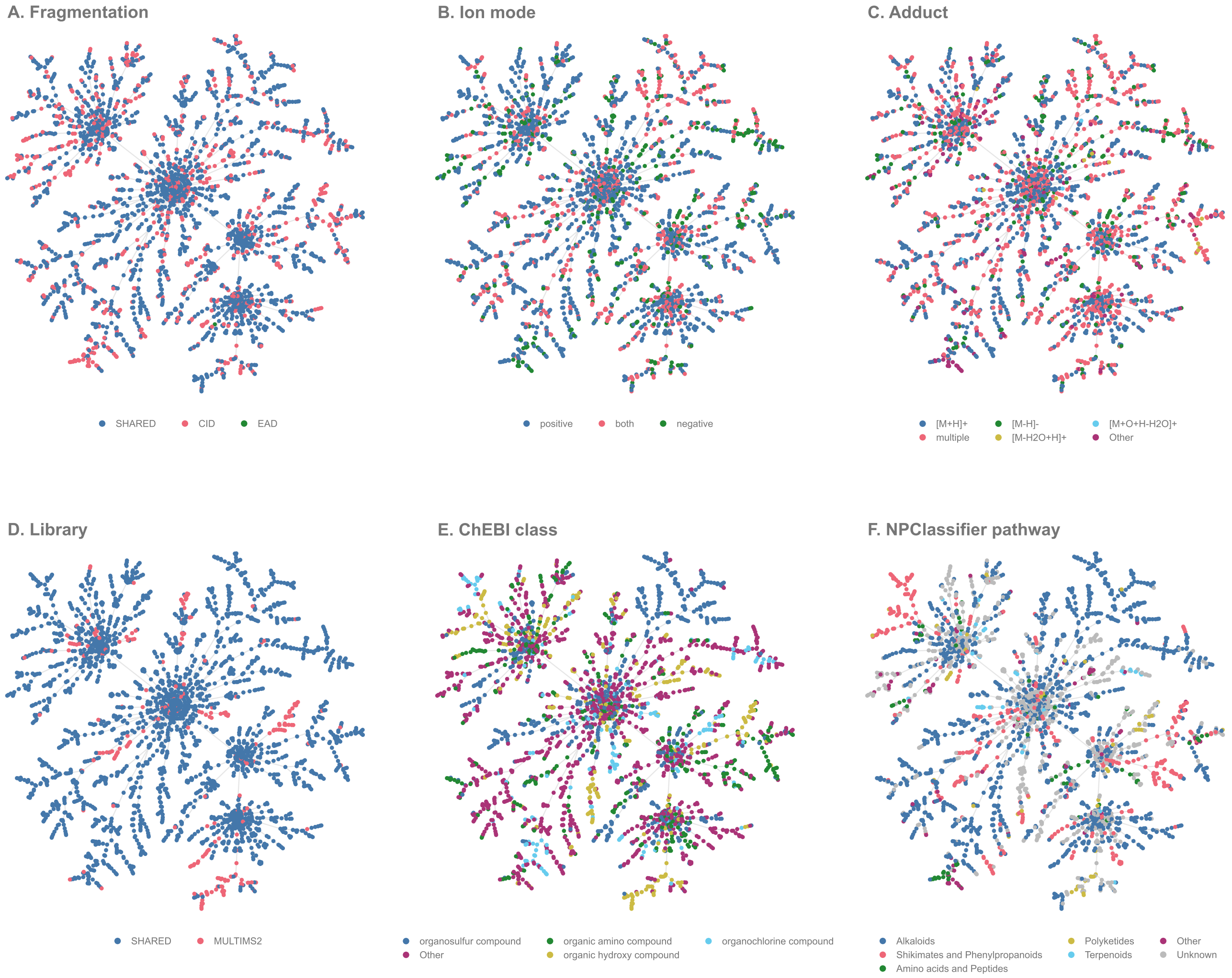

Figure 3. Chemical space coverage of the MultiMS^2 library
Each node represents a unique compound; layout reflects structural similarity based on MAP4 fingerprints [18] embedded via MinHash LSH and visualized with TMAP [19]. The same layout is colored by six metadata dimensions. The upper row represents metadata from the dataset itself while the lower one represents external metadata. An interactive version is available at `https://github.com/zamboni-lab/MultiMS2/figures/tmap.html`.
Panel A: Fragmentation mode. CID-only, EAD-only, and shared modalities, with the majority of the compounds acquired in both modalities.
Panel B: Ionization mode. Positive-only, negative-only, and compounds detected in both modes.
Panel C: Adduct type. The dominant adduct *[M+H]*$^+$ accounts for most positive-mode entries; *[M-H]*$^-$ dominates negative mode; but a great variety of less common adducts is also visible.
Panel D: Spectral libraries. Comparison with openly accessible external spectral libraries. Some clustered areas were previously not covered, confirming that MultiMS^2 provides substantial novel coverage.
Panel E: ChEBI chemical class [20]. Showing the chemical diversity of the library.
Panel F: NPClassifier biosynthetic pathway [21]. Alkaloids, shikimates and phenylpropanoids, amino acids and peptides, polyketides, and terpenoids are well represented.

- **Energy-aware modeling**: Incorporating collision energy as a continuous variable to improve spectral simulation.
- **Cross-dissociation transfer learning**: Leveraging knowledge from one fragmentation technique to improve performance on another.

The dataset also serves as a benchmark for model robustness, enabling direct comparison of algorithm performance across different fragmentation techniques.

Beyond machine learning, MultiMS^2 can be integrated into existing spectral matching platforms, enhancing annotation confidence through multi-modal spectral libraries. It supports workflows in environmental screening, clinical metabolomics, and systems biology.

Availability of source code and requirements

- Project name: MultiMS^2
- Project repository: `https://github.com/zamboni-lab/MultiMS2`
- Operating system(s): Platform independent (Docker container provided)
- Programming language:
  Python Programming Language (RRID:SCR_008394)
  R Project for Statistical Computing (RRID:SCR_001905)
  Bash (RRID:SCR_021268)
- Other requirements:
  Docker Desktop (RRID:SCR_016445)
  ProteoWizard (RRID:SCR_012056)
  mzmine (RRID:SCR_012040)
  uv (`https://docs.astral.sh/uv/`)
- License: MIT License
- Any restrictions to use by non-academics: None

Data availability

The data sets supporting the results of this article are available in both Zenodo [22] and MassIVE repositories [23] under permissive CC0 1.0 Universal License. Because of size limitations, the library was split into five partitions on GNPS:

- Partition 1
- Partition 2
- Partition 3
- Partition 4
- Partition 5

Declarations

List of abbreviations

- **CID**: Collision-Induced Dissociation
- **EAD**: Electron-Activated Dissociation
- **InChI(Key)**: International Chemical Identifier (Key)
- **MassIVE**: Mass Spectrometry Interactive Virtual Environment
- **SELFIES**: Self-Referencing Embedded Strings
- **SMILES**: Simplified Molecular Input Line Entry System

Ethical Approval

Not applicable

Consent for publication

Not applicable

Competing Interests

The authors declare that they have no competing interests.

Funding

This work was supported by grants from the Swiss National Science Foundation (project MetabolinkAI, #10002786), ETH Zurich (23-2 ETH-037), and the Strategic Focal Area Personalized Health and Related Technologies (PHRT) of the ETH Domain (#603).

Author's Contributions

Conceptualization: A.R. and N.Z. Data curation: A.R. Formal analysis: A.R. Funding acquisition: N.Z. Investigation: M.S.P.C. Methodology: A.R. and N.Z. Project administration: N.Z. Resources: N.Z. Software: A.R. Supervision: N.Z. Validation: A.R. and M.S.P.C. Visualization: A.R. Writing-original draft: A.R. Writing-review and editing: A.R. and N.Z.

Acknowledgements

The authors acknowledge Jasmine Zemlin (orcid:0000-0003-0713-9956), Yasin El Abiead (orcid:0000-0003-4392-7706), and Mingxun Wang (orcid:0000-0001-7647-6097) for their help with the GNPS library creation and sharing.

The authors used AI-assisted language models solely for language editing and clarity improvement. No scientific content, data analysis, or conclusions were generated by the tool. All outputs were critically reviewed by the authors.

References

1. Wang M, Carver JJ, Phelan VV, Sanchez LM, Garg N, Peng Y, et al. Sharing and community curation of mass spectrometry data with Global Natural Products Social Molecular Networking. Nature Biotechnology 2016 Aug;34(8):828–837. http://dx.doi.org/10.1038/nbt.3597.
2. Neumann S, Meier R, Wenk M, Elapavalore A, Nishioka T, Schulze T, et al. MassBank: an open and FAIR mass spectral data resource. Nucleic Acids Research 2025 Nov;http://dx.doi.org/10.1093/nar/gkaf1193.
3. de Jonge NF, Hecht H, Strobel M, Wang M, van der Hooft JJJ, Huber F. Reproducible MS/MS library cleaning pipeline in matchms. Journal of Cheminformatics 2024 Jul;16(1). http://dx.doi.org/10.1186/s13321-024-00878-1.
4. Gupta V, Qiang H, Chung HH, Herbst E, Skinnider M. Comprehensive curation and harmonization of small molecule MS/MS libraries in Spectraverse 2025 Oct;http://dx.doi.org/10.26434/chemrxiv-2025-4pzxn.
5. Kong F, Keshet U, Shen T, Rodriguez E, Fiehn O. LibGen: Generating High Quality Spectral Libraries of Natural Products for EAD-, UVPD-, and HCD-High Resolution Mass Spectrometers. Analytical Chemistry 2023 Nov;95(46):16810–16818. http://dx.doi.org/10.1021/acs.analchem.3c02263.
6. Brungs C, Schmid R, Heuckeroth S, Mazumdar A, Drexler M, Šácha P, et al. MSnLib: efficient generation of open multistage fragmentation mass spectral libraries. Nature Methods 2025 Sep;22(10):2028–2031. http://dx.doi.org/10.1038/s41592-025-02813-0.
7. Singh Y, Norris PC, Maharjan S, Gillespie J, Ferrante C, Ibrahim Z, et al. CIeaD: A Complementary CID and EAD Mass Spectral Library for Phytochemicals. Journal of the American Society for Mass Spectrometry 2025 Dec;http://dx.doi.org/10.1021/jasms.5c00329.
8. Smith CA, Maille GO, Want EJ, Qin C, Trauger SA, Brandon TR, et al. METLIN: A Metabolite Mass Spectral Database. Therapeutic Drug Monitoring 2005 Dec;27(6):747–751. http://dx.doi.org/10.1097/01.ftd.0000179845.53213.39.
9. Rutz A, Rainer J, CentroidR: Repository to centroid profile spectra. Zenodo; 2025. https://zenodo.org/doi/10.5281/zenodo.17250307.
10. Rutz A, Povoa Correia MS, Zamboni N, MultiMS2 spectral library - MGF and processing workflow. Zenodo; 2025. https://zenodo.org/doi/10.5281/zenodo.17417089.
11. Weininger D. SMILES, a chemical language and information system. 1. Introduction to methodology and encoding rules. Journal of Chemical Information and Computer Sciences 1988 Feb;28(1):31–36. http://dx.doi.org/10.1021/ci00057a005.
12. Heller SR, McNaught A, Pletnev I, Stein S, Tchekhovskoi D. InChI, the IUPAC International Chemical Identifier. Journal of Cheminformatics 2015 May;7(1). http://dx.doi.org/10.1186/s13321-015-0068-4.
13. Krenn M, Häse F, Nigam A, Friederich P, Aspuru-Guzik A. Self-referencing embedded strings (SELFIES): A 100 Machine Learning: Science and Technology 2020 Oct;1(4):045024. http://dx.doi.org/10.1088/2632-2153/aba947.
14. Rutz A, GitHub - zamboni-lab/MultiMS2: A Multi-Modal, Multi-Energy MS2 Spectral Library — github.com;. https://github.com/zamboni-lab/MultiMS2, [Accessed 12-02-2026].
15. International Chemical Identifier - Wikipedia — en.wikipedia.org;. https://en.wikipedia.org/wiki/International_Chemical_Identifier#InChIKey, [Accessed 12-02-2026].
16. Karasawa K, Duchoslav E, Baba T. Fast Electron Detachment Dissociation of Oligonucleotides in Electron-Nitrogen Plasma Stored in Magneto Radio-Frequency Ion Traps. Analytical Chemistry 2022 Oct;94(44):15510–15517. http://dx.doi.org/10.1021/acs.analchem.2c04027.

17. Xing S, Shen S, Xu B, Li X, Huan T. BUDDY: molecular formula discovery via bottom-up MS/MS interrogation. Nature Methods 2023 Apr;20(6):881–890. http://dx.doi.org/10.1038/s41592-023-01850-x.
18. Capecchi A, Probst D, Reymond JL. One molecular fingerprint to rule them all: drugs, biomolecules, and the metabolome. Journal of Cheminformatics 2020 Jun;12(1). http://dx.doi.org/10.1186/s13321-020-00445-4.
19. Probst D, Reymond JL. Visualization of very large high-dimensional data sets as minimum spanning trees. Journal of Cheminformatics 2020 Feb;12(1). http://dx.doi.org/10.1186/s13321-020-0416-x.
20. Glauer M, Neuhaus F, Flügel S, Wosny M, Mossakowski T, Memariani A, et al. Chebifier: automating semantic classification in ChEBI to accelerate data-driven discovery. Digital Discovery 2024;3(5):896–907. http://dx.doi.org/10.1039/D3DD00238A.
21. Kim HW, Wang M, Leber CA, Nothias LF, Reher R, Kang KB, et al. NPClassifier: A Deep Neural Network-Based Structural Classification Tool for Natural Products. Journal of Natural Products 2021 Oct;84(11):2795–2807. http://dx.doi.org/10.1021/acs.jnatprod.1c00399.
22. Povoa Correia MS, Rutz A, Zamboni N, MultiMS2 spectral library - mzml positive and negative. Zenodo; 2025. https://zenodo.org/doi/10.5281/zenodo.17250693.
23. Zamboni N, MassIVE MSV000099369 - GNPS - MultiMS2 spectral library. MassIVE; 2025. https://massive.ucsd.edu/ProteoSAFe/dataset.jsp?accession=MSV000099369.

Figure 1
Click here to access/download;Figure;upset_main.pdf

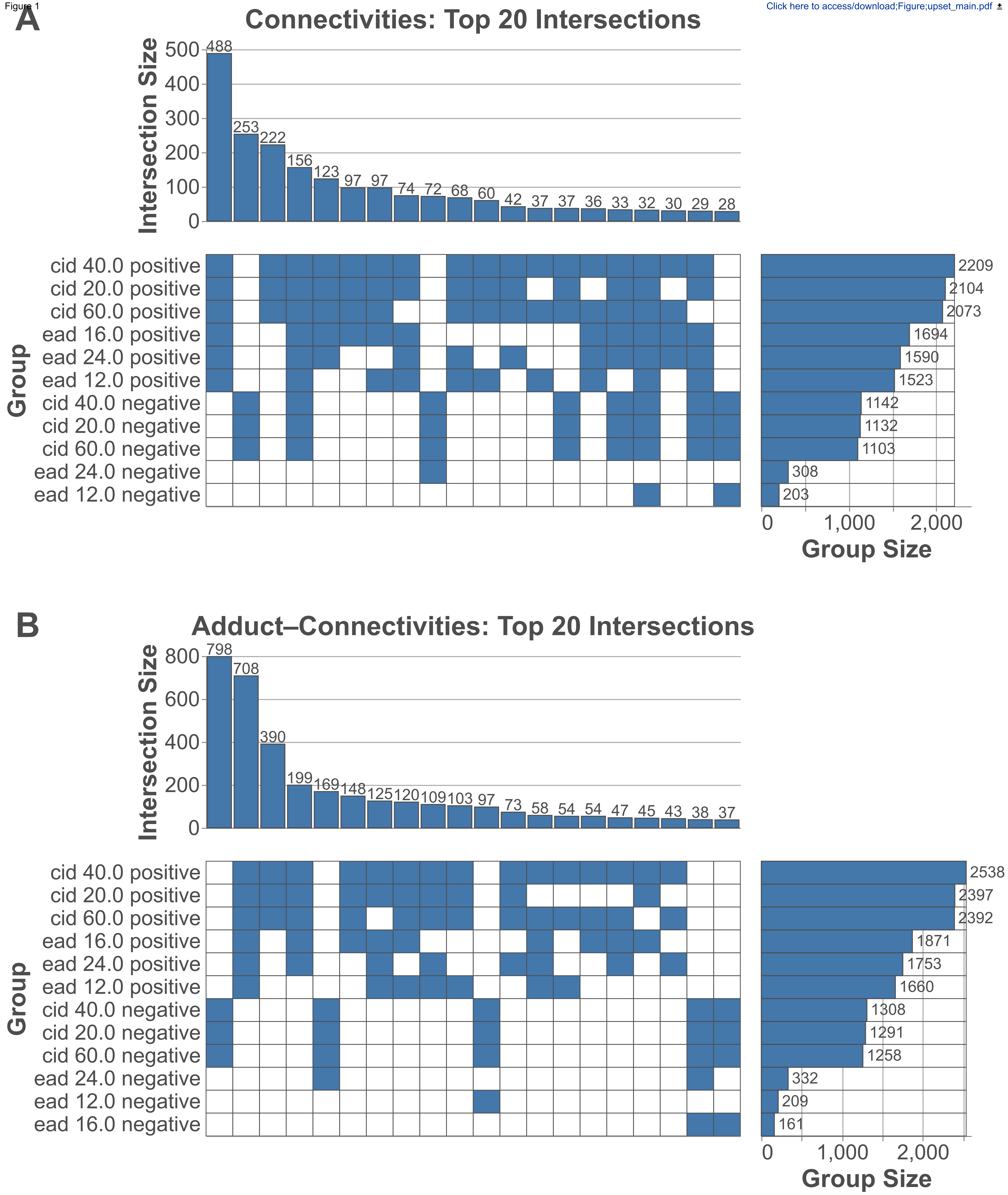

Click here to access/download;Figure;msbuddy_main.pdf

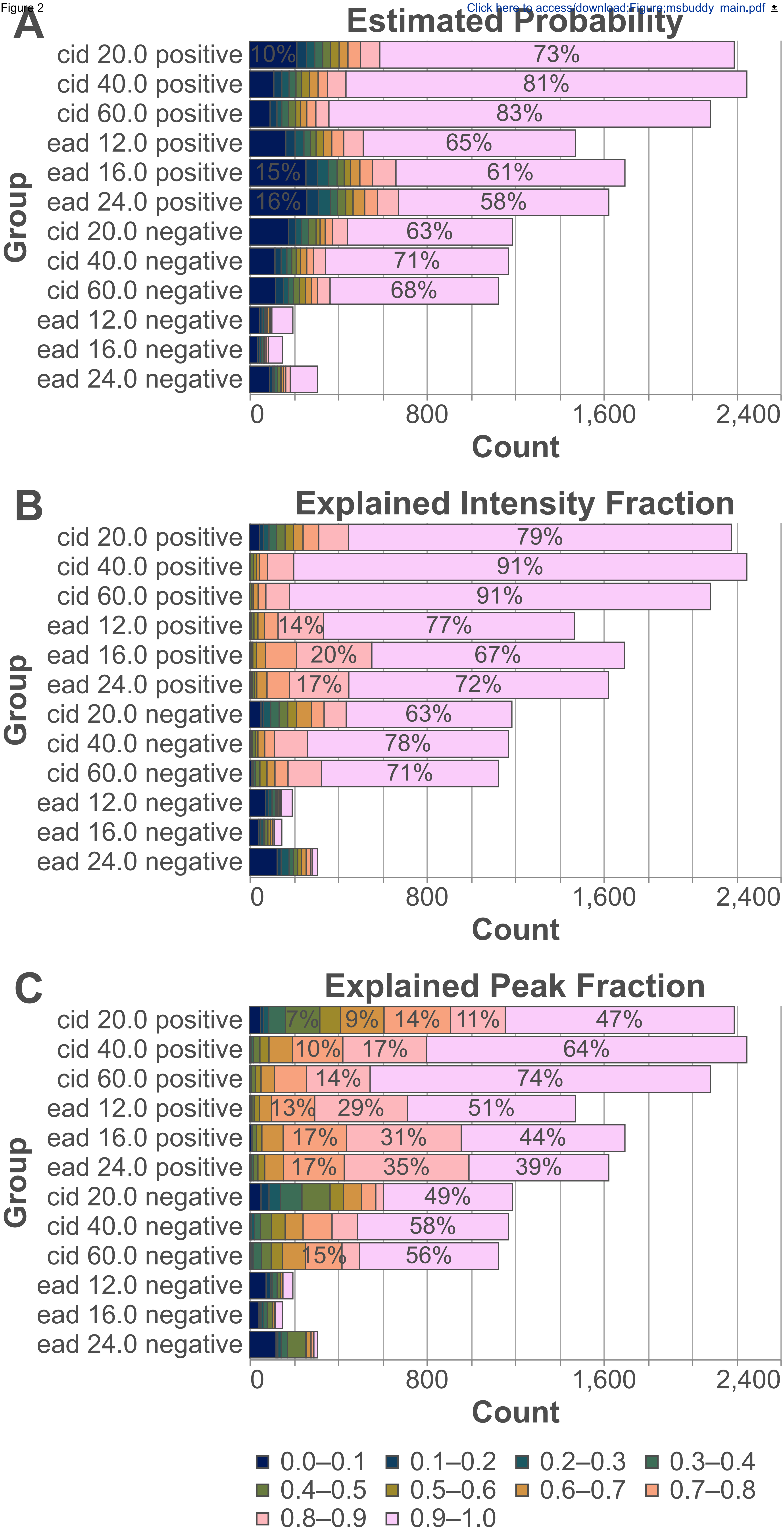

Figure 3

Click here to access/download;Figure;tmap.pdf

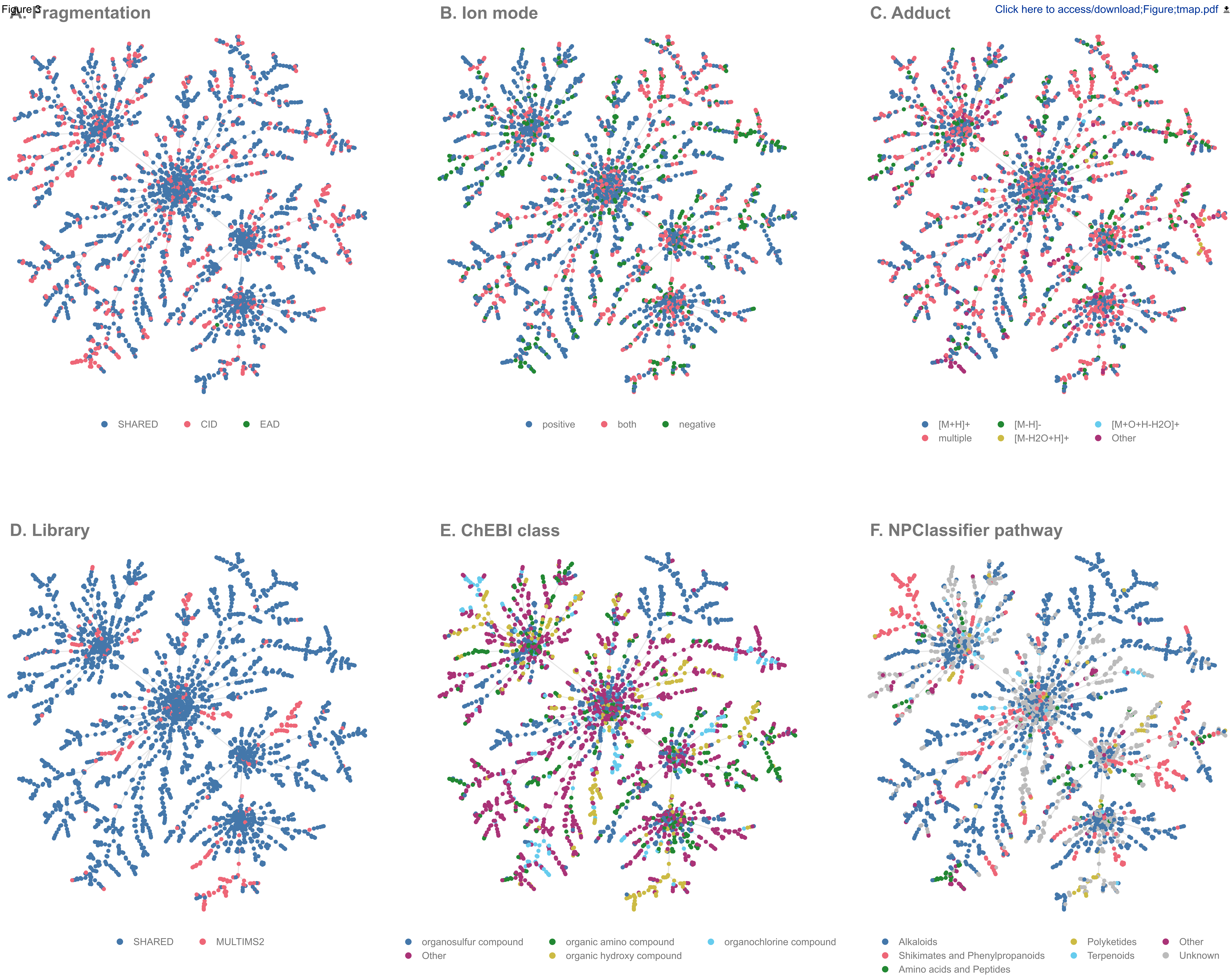

Supplement: giag069_GIGA-D-25-00518_revision_1 [file giag069_giga-d-25-00518_revision_1.pdf]
