# Supplementary material for: MultiMS2: A curated multi-modal, multi-energy spectral library for metabolomics
Source: Gigascience. 2026 Jun 10;15:giag069. doi: 10.1093/gigascience/giag069 (PMC13312951; doi:10.1093/gigascience/giag069)
Supplement: giag069_Authors_Response_To_Reviewer_Comments_original_submission [file giag069_authors_response_to_reviewer_comments_original_submission.pdf]

Author's Response To Reviewer Comments

Reviewer #1

> The manuscript has described MultiMS2: ACuratedMulti-Modal,Multi-Energy Spectral Library for Metabolomics. However, there are several key aspects that need to be clarified and further verified to enhance the scientific rigor and impact of the research. I recommend accepting the paper after minor revisions.

1. EAD energies are 12, 16, 24 eV, what is the consideration for the large span between the second and third energies?

We were hoping to capture possibly diverse dissociation events.
12 and 16 eV cover the range that has been previously reported to be optimal for lipids, bile acids, steroids and others.
Rather staying in the same range, we hypothesized tha higher energies might help to increase the yield of different types of fragments, as we saw for e.g. lipids.
Given the chemodiversity of our collection.
In addition, high kinetic energy are necessary for the activation of anions in negative mode ionization (see next point).
Hence, 24 was more appropriate to complement 12 and 16 eV.

2. The quality of EAD data in negative ion mode is far worse than that of other modes, and whether it is necessary to discuss it separately.

The lower number of fragments obtained by EAD in negative mode is not an indicator of poor quality, but the direct consequence of the electrostatic repulsion between the anionic compounds and electrons.
The lower coverage in negative mode is an expected, physical limitation of electron activation methods.
This is well-established in the EAD/ECD literature.

3. Since the results in figure 2 show that the interpretability of EAD data is worse, how to achieve the prediction based on EAD data mentioned by the author later.

We respectfully disagree with the Reviewer's claim that Figure 2 shows "interpretability of EAD data is worse".
We assessed formula assignment as a first-pass quality metric.
This refers to the composition of fragments and de novo formula prediction from MS2.
In contrast, it reveals nothing about structural elucidation capabilities, which may also well be superior for EAD.
More in general, we would like to stress that EAD and CID should no be antagonized.
The fundamental question is how the two modalities synergize.
Given the broad availability of CID spectra and CID-specific AI tools, we think that also in the future CID will be used in a first pass to infer the molecular formula and obtain a shortlist of putative candidates.
EAD could then be adopted in a second pass to rerank shortlisted compounds, exploiting EAD-specific fragments.

4. The necessity of using EAD-MS/MS has been repeatedly emphasized in the introduction and abstract, but the unique advantages and application scenarios of EAD-MS/MS are not reflected in the results.

We agree with the Reviewer and acknowledge this gap, but this is what a "Gigascience Data Note" is about: providing non-existing, high-quality data to the community to enable future studies that can systematically evaluate EAD advantages
We kept the comments on the advantages of EAD-MS/MS because they refer to previous studies that focused on specific classes.
We don't make any specific claims about this dataset.

5. "Multidimensional Mass Spectral Similarity Algorithm: Discriminate Disaccharide and Flavonoid Isomers Coupled with Online Energy-Resolved Acquisition and Electron Activation Dissociation Anal. Chem. 2025, 97, 26781−26792; Energy-resolved in-source dissociation spectra by tunable pulsed arc plasma ionization for the differentiation of structural isomers Chinese Chemical Letters https://doi.org/10.1016/j.cclet.2025.112060" should be cited.

We don't understand where this work should be cited and for what reason.
The first paper indeed used EAD on either disaccharides or flavonoids, but the it focuses on a modified spectrum similarity metrics. They don't make any spectrum available (other than in a low res PDF). This seems unrelated for the generic used of EAD, which doesn't justify a citation, for the same reason we don't cite dozens of other EAD papers.

The second paper is even less related: in-source fragmentation by plasma? it's from the same lab as above, but we struggle to see any link to our own contribution. There is no EAD, no spectral library. Please provide more guidance on where and why these two papers should be cited.

Reviewer #2

> Overall the MultiMs2 address a gap in EAD spectral library. The library is only shared as raw data files which limits its reuse as reference library. The major concern is negative mode datasets which is far poor coverage than positive mode datasets. This is contrary to endogenous metabolomics literature where negative mode outperforms positive mode. This suggests some major drawback in data acquisition or analysis methodology.

1. Is there any stat or reference available about breakdown of CID vs EAD spectral data in public libraries?

The short answer is no. It's hard to systematically search for EAD in repositories such as MoNA, GNPS, etc. because EAD is not reported in a systemstic way in the records. There is a lack of standardization.
We have to rely on primary literature, and the only sizeable EAD library appears to be the one published by the Fiehn lab in their LibGen publication (and more recently CIeaD, but it lacks structural identifiers).
This is symptomatic for the difficulty of obtaining solid stats.
This context strengthens the rationale for MultiMS2 as a resource.

2. METLIN and some other spectral libraries are exception to this which can be acknowledged

We rephrased to "these resources typically lack systematic coverage across dissociation mechanisms and multiple energies" to stress that the main limitation is multiple dissociatin mechanisms.
We are not aware that METLIN or other libraries provide multi-energy EAD spectra for metabolites, but this might be due to the difficulty obtaining the information (see #1).
If the reviewwer is aware of any accessible and citable source, we would be glad to correct the manuscript.

3. MedChem express website suggests Human Endogenous Metabolite Compound Library is consists of pre-disolved solutions of 825 metabolites
"Human Endogenous Metabolite Compound Library (1,000 standards; Selleck Chemicals, Art. No. L4500), which was pooled in sets of 10 compounds and diluted with 10% (v/v) Ethanol to a final concentration of 10 μM for injection."

We confirm the correctness of the statement.
At the time of purchase of L4500, we received 1013 compounds distributed across 13 96 WPs, all with associated metadata.
It seems that in the meantime, the number was reduced to 840.
Importantly, this does not affect any of our data, analyses, or conclusions.

4. The practical challenge in preparation of these compounds is solubility in single solvent or solvent mixtures (10% ethanol may not be accurate). Can authors discuss or cite how they addressed such issues?

Also, the mixture of 10 compounds is based on non-overlapping precursor masses?

We thank the reviewer for raising this practical point.
We have added a clarifying note to the Methods section.
Compounds were used as supplied by the manufacturer (pre-dissolved) or prepared following manufacturer protocols.
Compound pooling was designed to maximize throughput; pools were assembled to minimize precursor mass overlap, and any remaining conflicts were resolved during data processing, consistent with standard practice in large-scale spectral library acquisition workflows.

5. For direct injection buffered mobile phase with formic acid may not be adequate for negative ionization mode. This may contribute to the poor performance of negative mode ionization datasets. Authors strongly recommended using ammonium hydroxide, or other basic modifiers for negative mode ionization.

We thank the reviewer for this constructive observation.
We have added a note to the manuscript acknowledging that the use of formic acid as a mobile phase modifier, while standard for positive mode ESI, is suboptimal for negative mode ionization.
Future work and library and updates will explore the use of alkaline modifiers such as ammonium hydroxide to improve negative mode coverage.

6. What is rational for selecting 3 CEs?

We chose 3 energies for practical reasons.
We had to acquire all MS2 spectra of several compounds incl. multiple adducts during the apex of the elution peak.
Because of limitation in the acquisition software, we had to use DDA, which lead to the fragmentation of background ions.
The DDA engine has a powerful dynamic background substraction that prevents to select constant signals, but it only works in combination with a chromatographic separation.
Since we used flow injection, the dynamic background substraction wasn't able to distinguish analytes of interest from background, and had to be deactivated.
We partially mitigated the problem with a static exclusion list, but still had to take into account fragmenting unwanted precursors before DDA selected the adducts of interest.
The selection of three collision energies follows the approach established by other spectral libraries, which demonstrated that coverage of low, medium, and high fragmentation regimes captures complementary structural information without excessive redundancy.
Once the number was set, we picked 3 energy values to span the whole range that we typically use.
Note that the baseline kinetic energy in TOFMS mode is 10 eV.

7. What is biochemical pathway and/or chemical class specific breakdown of the presented compounds?

This is a fair and valuable question.
We have generated a chemical class and pathway annotation map using TMAP, which has been deposited in the repository alongside the spectral data.
A reference to this visualization has been added to the manuscript, allowing users to rapidly assess the chemical space covered by MultiMS2.

8. Figure 1 is clearly presented and only highlights poor coverage for negative mode datasets. This contradicts with literature and general observation about endogenous metabolites where negative mode outperforms positive mode. Again, authors should revisit their data acquisition and processing strategy to address this concern.

We respectfully note that the composition of the library reflects the ionization properties of the commercially available standards used, which are enriched in compounds that preferentially ionize in positive mode.
This is consistent with observations reported for comparable spectral libraries (e.g., Brungs et al., MSnlib).
The observed positive-mode enrichment is therefore a characteristic of the compound collection, not a

methodological failure.
Please note that the low numbers reported for EAD in negative mode are caused by well-known physical limitations of ExD, not by acquisition or processing.

9. Reuse and data availability: raw data is provided as files. This is appreciated, however, for reuse of the library for non-computational chemists this will cumbersome task to extract MS2 data and REUSE it as a reference library. It is recommended that library to be part of MassBank or MONA repositories in sharable MSP or MGF formats.

All spectra have been deposited in GNPS in MGF format, as described in the Data Availability section, and are additionally available on Zenodo.
Raw files are provided for full transparency and to enable reprocessing.
We believe this addresses the reviewer's concern.

10. Several key references of other spectral libraries are missing.

We thank the reviewer for this suggestion and have carefully reviewed the reference list.
We have incorporated additional citations to key spectral resources throughout the manuscript where most relevant.

Editorial Manager®

Cookie Preference Center

We use cookies which are necessary to make our site work. We may also use additional cookies to analyze, improve and personalize our content and your digital experience. For more information, see our Cookie Policy.

You may choose not to allow some types of cookies. However, blocking some types may impact your experience of our site and the services we are able to offer. See the different category headings below to find out more or change your settings.

Allow all

Manage Consent Preferences

Strictly Necessary Cookies

Always active

These cookies are necessary for the website to function and cannot be switched off in our systems. They are usually only set in response to actions made by you which

amount to a request for services, such as setting your privacy preferences, logging in or filling in forms. You can set your browser to block or alert you about these cookies, but some parts of the site will not then work.

Functional Cookies

These cookies enable the website to provide enhanced functionality and personalisation. They may be set by us or by third party providers whose services we have added to our pages. If you do not allow these cookies then some or all of these services may not function properly.

Performance Cookies

These cookies allow us to count visits and traffic sources so we can measure and improve the performance of our site. They help us to know which pages are the most and least popular and see how visitors move around the site.

Targeting Cookies

These cookies may be set through our site by our advertising partners. They may be used by those companies to build a profile of your interests and show you relevant adverts on other sites. If you do not allow these cookies, you will experience less targeted advertising.

Cookie List

Clear

Apply Cancel

Consent Leg.Interest

Confirm my choices

Powe
