# Supplementary material for: MultiMS2: A curated multi-modal, multi-energy spectral library for metabolomics
Source: Gigascience. 2026 Jun 10;15:giag069. doi: 10.1093/gigascience/giag069 (PMC13312951; doi:10.1093/gigascience/giag069)
Supplement: giag069_Authors_Response_To_Reviewer_Comments_revision_1 [file giag069_authors_response_to_reviewer_comments_revision_1.pdf]

Author's Response To Reviewer Comments

Reviewer #2: Authors should make respective improvements in the manuscript and not just providing rebuttal. To make this EAD dataset unique and complementary which is objective of this Giga science note, authors should make efforts to justify how it is better (other than scale) from earlier reports LibGen, CleaD etc libraries.

Regarding authors rebuttal about negative mode:
1. If compounds are not ionizing properly in negative mode due to formic acid, you never get sufficient precursor ions into the trap in the first place. Same issue is probably with LibGen data aquisition. It is Data Quality concern for negative mode and not just physics limitation.

2. The authors' statement that poor EAD negative mode coverage as"well-established physical limitations of ExD" is an overreach and borrowing a physics argument from peptide (multicharged) MS literature (which by default is positive ion mode), very few to none proteomicst studies use negative ionization mode, so applying it to small molecule is not fair (although there is merit with physics limitations).
3. Authors should make efforts to first improve ionization in negative mode and then provide evidence for poor EAD coverage in MS/MS data. This is not beyond the scope of this note as it claims tobe complementary resource for small molecules.
4. It still not clear how standards are prepared, if vendor protocols are no longer available, how should one can reproduce the preparations with known solubility issues.

Response: We thank the Reviewer for this comment. Size matters, but we also made an effort to entirely FAIRify the process (raw data, libraries, scripts), provide extensive quality controls (Figure 1), fully annotated MSP/MGFs, and a dscription of the chemical space (TMAPs etc). We don't want to criticize other sources, but LibGen offers only ~300 EAD spectra (hard to find on MoNA), and CleaD doesn't not include chemical identifiers, adducts, ... basically anything that makes the resource FAIR.

Regarding the reviewer's comments on negative ion mode:

1. Reviewer comment: If compounds are not ionizing properly in negative mode due to formic acid, you never get sufficient precursor ions into the trap in the first place. The same issue is probably present in LibGen data acquisition. This is a data-quality concern for negative mode and not just a physics limitation.

Response: We respectfully disagree that insufficient ionization is the primary explanation for the lower recovery of negative-mode EAD spectra. The negative-mode CID data, acquired under identical ionization conditions, show that precursor ions were generated and isolated sufficiently, as presented in Figure 1A–C. This indicates that the issue is not primarily poor ionization, but rather the low MS/MS yield observed for negative-mode EAD.

2. Reviewer comment: The authors' statement that poor EAD negative-mode coverage reflects "well-established physical limitations of ExD" is an overreach and appears to borrow a physics argument from peptide, multicharged MS literature, which is by default positive-ion mode. Very few proteomics studies use negative ionization mode, so applying this argument to small molecules is not fair, although there is merit to the physics limitation.

Response: The literature we refer to is not from peptides, but from anionic oligonucleotides, such as 10.1016/j.jasms.2010.02.025, 10.1021/acs.analchem.2c04027. Citing from the latter work "the intense electron beam did not increase EDD efficiency dramatically in the reported electron beam energy range: 18–24 eV because the electron beam and the precursor anions were still repulsive". The same issue applies to smaller, anionic compounds, which explains why negative mode EAD is never really used. As an example, we refer to EAD studies on acylcarnitines (10.1007/s00216-025-06234-y) or on eicosanoids (10.1021/jasms.2c00256), in which the positive protonated and sodiated forms are always preferred over the negative anions.

3. Reviewer comment: Authors should make efforts to first improve ionization in negative mode and then provide evidence for poor EAD coverage in MS/MS data. This is not beyond the scope of this note as it claims to be a complementary resource for small molecules.

Response: We appreciate the reviewer's concern. However, based on the available data, we do not find evidence that ionization is the limiting factor. As noted above, the negative-mode CID spectra acquired under the same ionization conditions demonstrate that sufficient precursor ions were available. The lower recovery is therefore more consistent with reduced EAD MS/MS yield in negative ion mode.

We also respectfully note that the primary objective of this Data Note is to provide a unique, FAIR, and high-quality EAD spectral resource for small molecules. We are confident to meet all these criteria. Although the negative-mode EAD subset is smaller, its size does not define the overall value of the resource. Indeed, the dataset would remain a valuable and complementary EAD resource even without the negative-mode EAD spectra. In fact, the dataset is already being used by colleagues for spectral annotation and for training machine-learning and AI models.

We are in the process of acquiring data for 24'000 pure standards, and negotiating to expand this effort > 100'000 compounds. However, the scope and content of the first version of the dataset are now fixed.

4. Reviewer comment: It is still not clear how standards are prepared. If vendor protocols are no longer available, how can one reproduce the preparations, especially given known solubility issues?

Response: We thank the reviewer for pointing out that this needed to be clearer. The standard preparation procedure is described in the Experimental Methods section.

Editorial Manager®

Cookie Preference Center

We use cookies which are necessary to make our site work. We may also use additional cookies to analyze, improve and personalize our content and your digital experience. For more information, see our Cookie Policy.

You may choose not to allow some types of cookies. However, blocking some types may impact your experience of our site and the services we are able to offer. See the different category headings below to find out more or change your settings.

Allow all

Manage Consent Preferences

Strictly Necessary Cookies

Always active

These cookies are necessary for the website to function and cannot be switched off in our systems. They are usually only set in response to actions made by you which amount to a request for services, such as setting your privacy preferences, logging in or filling in forms. You can set your browser to block or alert you about these cookies, but some parts of the site will not then work.

Functional Cookies

These cookies enable the website to provide enhanced functionality and personalisation. They may be set by us or by third party providers whose services we have added to our pages. If you do not allow these cookies then some or all of these services may not function properly.

Performance Cookies

These cookies allow us to count visits and traffic sources so we can measure and improve the performance of our site. They help us to know which pages are the most and least popular and see how visitors move around the site.

Targeting Cookies

These cookies may be set through our site by our advertising partners. They may be used by those companies to build a profile of your interests and show you relevant adverts on other sites. If you do not allow these cookies, you will experience less targeted advertising.

Cookie List

Clear

Apply Cancel

Consent Leg.Interest

Confirm my choices

Power[illegible]
