# Supplementary material for: MultiMS2: A curated multi-modal, multi-energy spectral library for metabolomics
Source: Gigascience. 2026 Jun 10;15:giag069. doi: 10.1093/gigascience/giag069 (PMC13312951; doi:10.1093/gigascience/giag069)
Supplement: giag069_Reviewer_1_Report_original_submission — Reviewer 1 -- 1/13/2026 [file giag069_reviewer_1_report_original_submission.pdf]

Reviewer Report

Title: MultiMS2: A Curated Multi-Modal, Multi-Energy Spectral Library for Metabolomics

Version: Original Submission **Date:** 1/13/2026

Reviewer name: Reviewer 1

Reviewer Comments to Author:

The manuscript has described MultiMS2: ACuratedMulti-Modal,Multi-Energy Spectral Library for Metabolomics. However, there are several key aspects that need to be clarified and further verified to enhance the scientific rigor and impact of the research. I recommend accepting the paper after minor revisions.

1. EAD energies are 12, 16, 24 eV, what is the consideration for the large span between the second and third energies?
2. The quality of EAD data in negative ion mode is far worse than that of other modes, and whether it is necessary to discuss it separately.
3. Since the results in figure 2 show that the interpretability of EAD data is worse, how to achieve the prediction based on EAD data mentioned by the author later.
4. The necessity of using EAD-MS/MS has been repeatedly emphasized in the introduction and abstract, but the unique advantages and application scenarios of EAD-MS/MS are not reflected in the results.
5. "Multidimensional Mass Spectral Similarity Algorithm: Discriminate Disaccharide and Flavonoid Isomers Coupled with Online Energy-Resolved Acquisition and Electron Activation Dissociation Anal. Chem. 2025, 97, 26781âˆ’26792; Energy-resolved in-source dissociation spectra by tunable pulsed arc plasma ionization for the differentiation of structural isomers Chinese Chemical Letters https://doi.org/10.1016/j.cclet.2025.112060" should be cited.

Methods

Are the methods appropriate to the aims of the study, are they well described, and are necessary controls included? Choose an item.

Conclusions

Are the conclusions adequately supported by the data shown? Choose an item.

Reporting Standards

Does the manuscript adhere to the journal's guidelines on [minimum standards of reporting?]() Choose an item.

Choose an item.

Statistics

Are you able to assess all statistics in the manuscript, including the appropriateness of statistical tests used? Choose an item.

Quality of Written English

Please indicate the quality of language in the manuscript: Choose an item.

Declaration of Competing Interests

Please complete a declaration of competing interests, considering the following questions:

- Have you in the past five years received reimbursements, fees, funding, or salary from an organisation that may in any way gain or lose financially from the publication of this manuscript, either now or in the future?
- Do you hold any stocks or shares in an organisation that may in any way gain or lose financially from the publication of this manuscript, either now or in the future?
- Do you hold or are you currently applying for any patents relating to the content of the manuscript?
- Have you received reimbursements, fees, funding, or salary from an organization that holds or has applied for patents relating to the content of the manuscript?
- Do you have any other financial competing interests?
- Do you have any non-financial competing interests in relation to this paper?

If you can answer no to all of the above, write 'I declare that I have no competing interests' below. If your reply is yes to any, please give details below.

I declare that I have no competing interests.

I agree to the open peer review policy of the journal. I understand that my name will be included on my report to the authors and, if the manuscript is accepted for publication, my named report including any attachments I upload will be posted on the website along with the authors' responses. I agree for my report to be made available under an Open Access Creative Commons CC-BY license (http://creativecommons.org/licenses/by/4.0/). I understand that any comments which I do not wish to be included in my named report can be included as confidential comments to the editors, which will not be published.

Choose an item.

To further support our reviewers, we have joined with Publons, where you can gain additional credit to further highlight your hard work (see: https://publons.com/journal/530/gigascience). On publication of this paper, your review will be automatically added to Publons, you can then choose whether or not to claim your Publons credit. I understand this statement.

Yes Choose an item.
