# Supplementary material for: MultiMS2: A curated multi-modal, multi-energy spectral library for metabolomics
Source: Gigascience. 2026 Jun 10;15:giag069. doi: 10.1093/gigascience/giag069 (PMC13312951; doi:10.1093/gigascience/giag069)
Supplement: giag069_Reviewer_2_Report_original_submission — Reviewer 2 -- 1/25/2026 [file giag069_reviewer_2_report_original_submission.pdf]

Reviewer Report

Title: MultiMS2: A Curated Multi-Modal, Multi-Energy Spectral Library for Metabolomics

Version: Original Submission **Date:** 1/25/2026

Reviewer name: Reviewer 2

Reviewer Comments to Author:

Overall the MultiMs2 address a gap in EAD spectral library. The library is only shared as raw data files which limits its reuse as reference library. The major concern is negative mode datasets which is far poor coverage than positive mode datasets. This is contrary to endogenous metabolomics literature where negative mode outperforms positive mode. This suggests some major drawback in data acquisition or analysis methodology.

1. Is there any stat or reference available about breakdown of CID vs EAD spectral data in public libraries?
"EAD spectra remain scarce in public repositories, restricting both manual interpretation"
2. METLIN and some other spectral libraries are exception to this which can be acknowledged
"these resources typically lack systematic coverage across multiple energies and dissociation mechanisms"
3. MedChem express website suggests Human Endogenous Metabolite Compound Library is consists of pre-disolved solutions of 825 metabolites
"Human Endogenous Metabolite Compound Library (1,000 standards; Selleck Chemicals, Art. No. L4500), which was pooled in sets of 10 compounds and diluted with 10% (v/v) Ethanol to a final concentration of 10 Î¼M for injection."
4. The practical challenge in preparation of these compounds is solubility in single solvent or solvent mixtures (10% ethanol may not be accurate). Can authors discuss or cite how they addressed such issues? Also, the mixture of 10 compounds is based on non-overlapping precursor masses?
5. For direct injection buffered mobile phase with formic acid may not be adequate for negative ionization mode. This may contribute to the poor performance of negative mode ionization datasets. Authors strongly recommended using ammonium hydroxide, or other basic modifiers for negative mode ionization.
6. What is rational for selecting 3 CEs?
7. What is biochemical pathway and/or chemical class specific breakdown of the presented compounds?
8. Figure 1 is clearly presented and only highlights poor coverage for negative mode datasets. This contradicts with literature and general observation about endogenous metabolites where negative mode outperforms positive mode. Again, authors should revisit their data acquisition and processing strategy to address this concern.
9. Reuse and data availability: raw data is provided as files. This is appreciated, however, for reuse of the library for non-computational chemists this will cumbersome task to extract MS2 data and REUSE it as a reference library. It is recommended that library to be part of MassBank or MONA repositories in sharable MSP or MGF formats.

10. Several key references of other spectral libraries are missing.

Methods

Are the methods appropriate to the aims of the study, are they well described, and are necessary controls included? Choose an item.

Conclusions

Are the conclusions adequately supported by the data shown? Choose an item.

Reporting Standards

Does the manuscript adhere to the journal's guidelines on minimum standards of reporting? Choose an item.

Choose an item.

Statistics

Are you able to assess all statistics in the manuscript, including the appropriateness of statistical tests used? Choose an item.

Quality of Written English

Please indicate the quality of language in the manuscript: Choose an item.

Declaration of Competing Interests

Please complete a declaration of competing interests, considering the following questions:

- Have you in the past five years received reimbursements, fees, funding, or salary from an organisation that may in any way gain or lose financially from the publication of this manuscript, either now or in the future?
- Do you hold any stocks or shares in an organisation that may in any way gain or lose financially from the publication of this manuscript, either now or in the future?
- Do you hold or are you currently applying for any patents relating to the content of the manuscript?
- Have you received reimbursements, fees, funding, or salary from an organization that holds or has applied for patents relating to the content of the manuscript?
- Do you have any other financial competing interests?
- Do you have any non-financial competing interests in relation to this paper?

If you can answer no to all of the above, write 'I declare that I have no competing interests' below. If your reply is yes to any, please give details below.

'I declare that I have no competing interests'

I agree to the open peer review policy of the journal. I understand that my name will be included on my report to the authors and, if the manuscript is accepted for publication, my named report including any attachments I upload will be posted on the website along with the authors' responses. I agree for my report to be made available under an Open Access Creative Commons CC-BY license (http://creativecommons.org/licenses/by/4.0/). I understand that any comments which I do not wish to be included in my named report can be included as confidential comments to the editors, which will not be published.

Choose an item.

To further support our reviewers, we have joined with Publons, where you can gain additional credit to further highlight your hard work (see: https://publons.com/journal/530/gigascience). On publication of this paper, your review will be automatically added to Publons, you can then choose whether or not to claim your Publons credit. I understand this statement.

Yes Choose an item.
