# Supplementary material for: MultiMS2: A curated multi-modal, multi-energy spectral library for metabolomics
Source: Gigascience. 2026 Jun 10;15:giag069. doi: 10.1093/gigascience/giag069 (PMC13312951; doi:10.1093/gigascience/giag069)
Supplement: giag069_Reviewer_2_Report_revision_1 — Reviewer 2 -- 4/27/2026 [file giag069_reviewer_2_report_revision_1.pdf]

Reviewer Report

Title: MultiMS2: A Curated Multi-Modal, Multi-Energy Spectral Library for Metabolomics

Version: Revision 1 **Date:** 4/27/2026

Reviewer name: Reviewer 2

Reviewer Comments to Author:

Authors should make respective improvements in the manuscript and not just providing rebuttal. To make this EAD dataset unique and complementary which is objective of this Giga science note, authors should make efforts to justify how it is better (other than scale) from earlier reports LibGen, CleaD etc libraries.

Regarding authors rebuttal about negative mode:

1. If compounds are not ionizing properly in negative mode due to formic acid, you never get sufficient precursor ions into the trap in the first place. Same issue is probably with LibGen data aquisition. It is Data Quality concern for negative mode and not just physics limitation.
2. The authors' statement that poor EAD negative mode coverage as"well-established physical limitations of ExD" is an overreach and borrowing a physics argument from peptide (multicharged) MS literature (which by default is positive ion mode), very few to none proteomicst studies use negative ionization mode, so applying it to small molecule is not fair (although there is merit with physics limitations).
3. Authors should make efforts to first improve ionization in negative mode and then provide evidence for poor EAD coverage in MS/MS data. This is not beyond the scope of this note as it claims tobe complementary resource for small molecules.
4. It still not clear how standards are prepared, if vendor protocols are no longer available, how should one can reproduce the preparations with known solubility issues.

Methods

Are the methods appropriate to the aims of the study, are they well described, and are necessary controls included? Choose an item.

Conclusions

Are the conclusions adequately supported by the data shown? Choose an item.

Reporting Standards

Does the manuscript adhere to the journal's guidelines on minimum standards of reporting? Choose an item.

Choose an item.

Statistics

Are you able to assess all statistics in the manuscript, including the appropriateness of statistical tests used? Choose an item.

Quality of Written English

Please indicate the quality of language in the manuscript: Choose an item.

Declaration of Competing Interests

Please complete a declaration of competing interests, considering the following questions:

- Have you in the past five years received reimbursements, fees, funding, or salary from an organisation that may in any way gain or lose financially from the publication of this manuscript, either now or in the future?
- Do you hold any stocks or shares in an organisation that may in any way gain or lose financially from the publication of this manuscript, either now or in the future?
- Do you hold or are you currently applying for any patents relating to the content of the manuscript?
- Have you received reimbursements, fees, funding, or salary from an organization that holds or has applied for patents relating to the content of the manuscript?
- Do you have any other financial competing interests?
- Do you have any non-financial competing interests in relation to this paper?

If you can answer no to all of the above, write 'I declare that I have no competing interests' below. If your reply is yes to any, please give details below.

I declare that I have no competing interests

I agree to the open peer review policy of the journal. I understand that my name will be included on my report to the authors and, if the manuscript is accepted for publication, my named report including any attachments I upload will be posted on the website along with the authors' responses. I agree for my report to be made available under an Open Access Creative Commons CC-BY license (http://creativecommons.org/licenses/by/4.0/). I understand that any comments which I do not wish to be included in my named report can be included as confidential comments to the editors, which will not be published.

I agree to the open peer review policy of the journal

To further support our reviewers, we have joined with Publons, where you can gain additional credit to further highlight your hard work (see: https://publons.com/journal/530/gigascience). On publication of this paper, your review will be automatically added to Publons, you can then choose whether or not to claim your Publons credit. I understand this statement.

Yes Choose an item.
